# Scholarly Context Adrift: Three out of Four URI References Lead to Changed Content

**DOI:** 10.1371/journal.pone.0167475

**Published:** 2016-12-02

**Authors:** Shawn M. Jones, Herbert Van de Sompel, Harihar Shankar, Martin Klein, Richard Tobin, Claire Grover

**Affiliations:** 1 Digital Library Research and Prototyping Team, Research Library, Los Alamos National Laboratory, Los Alamos, New Mexico, United States of America; 2 Language Technology Group, The University of Edinburgh, Edinburgh, Scotland, United Kingdom; University of Illinois-Chicago, UNITED STATES

## Abstract

Increasingly, scholarly articles contain URI references to “web at large” resources including project web sites, scholarly wikis, ontologies, online debates, presentations, blogs, and videos. Authors reference such resources to provide essential context for the research they report on. A reader who visits a web at large resource by following a URI reference in an article, some time after its publication, is led to believe that the resource’s content is representative of what the author originally referenced. However, due to the dynamic nature of the web, that may very well not be the case. We reuse a dataset from a previous study in which several authors of this paper were involved, and investigate to what extent the textual content of web at large resources referenced in a vast collection of Science, Technology, and Medicine (STM) articles published between 1997 and 2012 has remained stable since the publication of the referencing article. We do so in a two-step approach that relies on various well-established similarity measures to compare textual content. In a first step, we use 19 web archives to find snapshots of referenced web at large resources that have textual content that is representative of the state of the resource around the time of publication of the referencing paper. We find that representative snapshots exist for about 30% of all URI references. In a second step, we compare the textual content of representative snapshots with that of their live web counterparts. We find that for over 75% of references the content has drifted away from what it was when referenced. These results raise significant concerns regarding the long term integrity of the web-based scholarly record and call for the deployment of techniques to combat these problems.

## Introduction

Increasingly, scholarly papers reference **web at large** resources [1], that is, web resources that themselves are not scholarly papers but rather supporting resources including project web sites, scholarly wikis, ontologies, online debates, presentations, blogs, and videos. While these resources may not convey the scholarly essence of a paper, they do provide an important context pertaining to the research reported in the paper. After all, the author judged that it was relevant to include them, and, in the case of peer-reviewed papers, both reviewers and editors agreed by deciding to publish the paper with inclusion of the references to the web at large resources.

While scholarly papers are commonly referenced by means of persistent HTTP URIs that carry a Digital Object Identifier, web at large resources are referenced by means of regular HTTP URIs, which, from now on, are simply referred to as URIs. The Hiberlink project [2] coined the term **Reference rot** to denote the combination of two problems related to the use of HTTP URIs for referencing. Both problems are caused by the dynamic and ephemeral nature of the web:

- **Link rot**: The resource identified by a URI vanishes from the web. As a result, a URI reference to the resource ceases to provide access to referenced content.
- **Content drift**: The resource identified by a URI changes over time. The resource’s content evolves and can change to such an extent that it ceases to be representative of the content that was originally referenced.

As a result of reference rot, the context—made up of referenced web at large resources—that surrounds a paper may change over time. As such, a reader who looks up referenced resources some time after the publication of the referencing paper effectively explores the **current context** surrounding the paper, which may be significantly different from the **past context** that surrounded the paper at the time of its publication:

- The **past context** consists of the web at large resources referenced by a paper as they were at the time of publication of the referencing paper. If a link to a referenced resource still works when a reader follows it on the live web some time after the referencing paper was published, it is possible that the content at the end of the link is the same as it was at the time of publication. But, given content drift, the chances of this being the case diminish the further the consultation date is removed from the publication date.
- The **current context** is what a reader of a paper sees when following links to web at large resources on the live web. The current context may differ from what it was when the referencing paper was published because links may have rotted. In these cases, the reader will receive an error message as an explicit indicator that content that used to be there no longer is. But the current context may also differ because linked content has drifted. In these cases, when following the link, the reader may encounter content that significantly differs from the originally referenced content but has no means to assess whether or not that is the case.

When it comes to revisiting the past context of a paper, snapshots of linked resources in web archives—from now on referred to as **Mementos**—can come to the rescue. Web archives worldwide store hundreds of billions of Mementos and hence may coincidentally also store Mementos of referenced web at large resources. However, if such Mementos exist, the question arises as to what constitutes a **representative Memento**, a Memento that accurately reflects the referenced content as it was at the publication time of the referencing paper. Note that the publication time is chosen as an approximation for the time the paper’s author actually visited and referenced the resource, because consistent, machine-processable information for this latter time is unavailable.

**Fig 1:**
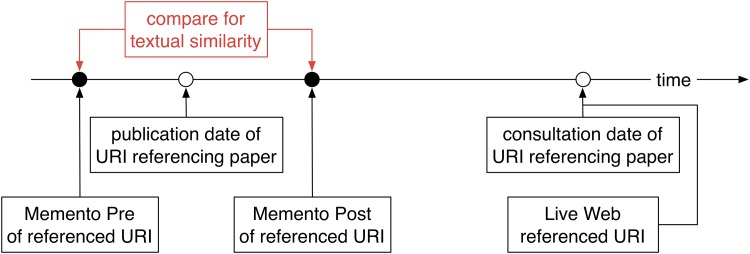
Past Context—Finding Representative Mementos.

**Fig 2:**
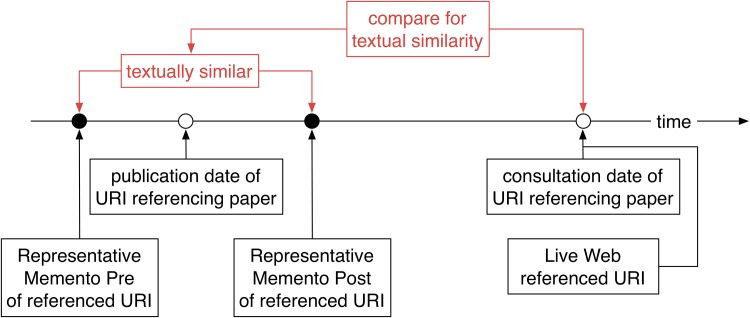
Current Context—Assessing Content Drift by Comparing a Representative Memento with the Associated Live Web Resource.

In this paper, we assess the extent of content drift for URI references to web at large resources that are made in STM articles. We do so by first making a quantitative assessment about the existence of representative Mementos for URI references to web at large resources. Once we have identified URI references for which representative Mementos exist, we proceed to assess the extent of content drift to which these references are subject. As such, this paper addresses two research questions. Tackling the first is a necessary step towards answering the second:

1. To what extent do representative Mementos exist for URI references to web at large resources (Fig 1)? For each URI reference, we poll multiple web archives in search of two Mementos: a Memento Pre that has a snapshot date closest and prior to the publication date of the referencing article, and a Memento Post that has a snapshot date closest and past the publication date. We then assess the similarity between these Pre and Post Mementos using a variety of similarity measures. Because these representative Mementos are used as the ground truth to answer the second research question, we use a high threshold to decide whether the Pre and Post Mementos are similar according to these measures. If they are, we decide that the Mementos are representative of the referenced content as it was around the time of publication of the referencing paper. We arrive at a novel insight into the extent to which the past context surrounding scholarly articles can be revisited.
2. What is the extent of content drift to which URI references to web at large resources are subject (Fig 2)? We use the resulting subset of all URI references for which representative Mementos exist and look up each URI on the live web. Predictably, and as shown by extensive prior link rot research, many URIs no longer exist. But, for those that still do, we use the same measures to assess the similarity between the representative Memento for the URI reference and its counterpart on the live web. We arrive at an unprecedented quantitative insight into the extent to which the current context, which surrounds a paper at consultation time, drifts away from the past context, which surrounded it at publication time.

**Fig 3:**
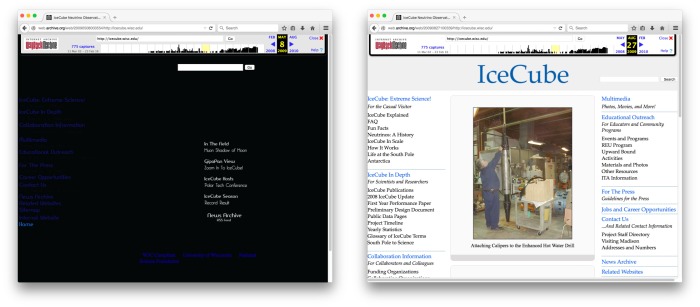
Significant Content Drift within a 3-Month Period.

**Fig 4:**
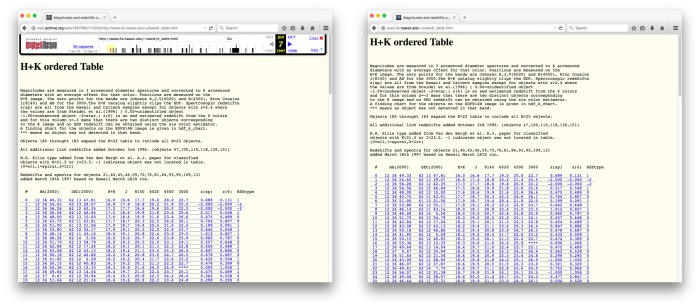
No Content Drift over a 19-Year Period.

To help explain the notion of content drift, we show two examples with snapshots of web pages that have changed over time (Fig 3) and that have not changed at all (Fig 4). A case of significant content drift is demonstrated by the homepage of the IceCube Neutrino Observatory at the University of Wisconsin. Its URI http://icecube.wisc.edu is referenced in [3] published on August 15 2009. The left part of Fig 3 shows a Memento from the Internet Archive with URI http://web.archive.org/web/20090508003554/http://icecube.wisc.edu/ and archival date May 8 2009. The right part shows a Memento, also from the Internet Archive, with URI http://web.archive.org/web/20090827100339/http://icecube.wisc.edu/ archived on August 27 2009. Both the content and the presentation have dramatically changed in the course of about three months. Note that the banners on the Mementos are dynamically inserted by the web archive and are not an integral part of the archived page.

On the other hand, Fig 4 shows a page of the Institute for Astronomy at the University of Hawaii with URI http://www.ifa.hawaii.edu/~cowie/k_table.html, referenced in [4] published on July 4 1997. The left part of Fig 4 shows a Memento from the Internet Archive with URI http://web.archive.org/web/19970607115534/http://www.ifa.hawaii.edu/~cowie/k_table.html that was archived on June 7 1997. The right part shows the live web version of that page as it was at the time of writing this paper. Note that the pages are identical; the content seemingly has not drifted a bit in 19 years.

## Related Work

Characterizing the frequency of change of web resources has been the subject of numerous research efforts in the past. Without exception, these studies support our intuition of the volatility of web at large resources. For example, Cho and Garcia-Molina [5] sought to develop improved web crawling strategies by studying the changes of web pages over time. They observed 270 web sites over the course of four months and determined that “more than 40% of pages in the *com* domain changed every day, while less than 10% of the pages in other domains changed at that frequency”. They also found that pages in *edu* and *gov* domains are more static. In a companion paper [6], they were able to determine that web pages change by a Poisson process, and suggest that the value of the HTTP response header *last-modified* is a better estimate of the frequency of change. In a related study from 2003, Fetterly et al. [7] crawled 151 million pages once a week for eleven weeks to determine the correlation between the change of web resources and factors such as document length. Their results show that longer documents changed more often and that the observed frequency of change is a good predictor of future changes. In a more recent study, Adar et al. [8] investigated the change of web content by crawling 55,000 web pages at hourly and sub-hourly periods and comparing the text between the same page at different times during the crawl. They found that a large portion of pages in their dataset changed more frequently than once per hourly. In order to optimize web crawlers they proposed focusing on the detection of “meaningful change” in web pages with the goal of avoiding the recrawl of pages that merely change based on non-content elements (e.g., embedded advertisements). In related work, Adar et al. [9] investigated the structural changes of web pages. They analyzed the change frequency of Document Object Model (DOM) elements within web pages and found that the median survival rate of DOM elements is 98% after one day, 95% after one week, 63% after five weeks, and only 11% after one year.

In addition to [1], link rot in scholarly literature was, for example, explored by Lawrence [10] in 2000, Casserly in 2002 [11], Sellitto in 2003 [12], McCown in 2004 [13], Falagas in 2007 [14], Duda in 2008 [15], Wagner in 2009 [16], and Moghaddam in 2010 [17]. These studies noted that the number of web at large resources cited in academic work was growing, but many of the referenced resources were no longer available on the live web. In 2011 Sanderson, Phillips, and Van de Sompel [18] analyzed the use of the Memento protocol [19] to find archived versions of resources referenced from arXiv and the institutional repository from the University of North Texas. They were principally concerned about the existence of Mementos for a referenced resource, but were also interested in the temporal delta between paper publication and the archiving of the referenced resource. These scholarly literature studies aimed at analyzing whether or not a live version of a referenced resource was still available and whether an archived version of the resource existed.

In addition to link rot, Jackson [20], Zittrain et al. [21] and Klein et al. [1] also investigated content drift of URI references. Jackson sampled 1,000 Memento URIs per year with archival dates between 2004 and 2013 from the UK web archive and checked their status on the live web. He found that after only two years, around 40% of the URIs were gone from the live web (link rot). He also found that the same ratio of URIs (40%) are “unrecognizably different” after two years (content drift). In aggregate, according to this study, 60% of URIs from the UK web archive corpus either suffer from link rot or content drift after only two years. Zittrain et al. manually evaluated all URI references found in U.S. Supreme court opinion papers starting in 1996. They analyzed the contexts in which the references occur and determined whether the originally intended content was still available at the live version of the URI. They found that around 50% of URI references suffered from content drift by 2014 when the study was conducted. Zittrain et al. also investigated reference rot in three law journals: Harvard Law Review (HLR), the Harvard Journal of Law and Technology (JOLT), and the Harvard Human Rights Journal (HRJ). Their corpus contained editions of the journals from 1999, 1996, and 1997, respectively, until mid 2012. Their study found that only 29.9% of the HRJ references, 26.8% of the HLR references, and 34.2% of the JOLT references contained the material originally cited. Hence between 65% and 73% of the URI references suffered from link rot or content drift. The study by Klein et al. extracted URI references from three vast scholarly corpora consisting of STM articles published between 1997 and 2012. They estimated the existence of representative Mementos for those URI references using an intuitive technique: if a Memento for a referenced URI existed with an archival datetime in a temporal window of 14 days prior and after the publication date of the referencing paper, the Memento was regarded representative. Using this ad-hoc technique, they found that representative Mementos existed for about 25% of URI references across the considered corpora. The study also assessed the extent of reference rot but did so at the aggregate level of journal articles instead of URI references, concluding that one out of five STM articles suffered from reference rot. In the work reported here, we make a quantitative assessment on the basis of text similarity measures of the existence of representative Mementos and the extent of content drift at the level of individual URI references.

Assessing the similarity of textual documents is a common problem in the web science and information retrieval realm. Multiple similarity measures have successfully been applied in the past across disciplines and research on the efficacy of these techniques exists. Though we are not engaging in improving web crawlers or document clustering, the success of the following work supports our choices of similarity measures.

Manku, Jain, and Sarma [22] evaluated the use of Charikar’s Simhash algorithm [23, 24] for web crawlers as a way to detect a recently-crawled page. After testing it during a crawl of eight billion web pages, they determined that Simhash was a very effective method of analyzing duplicate web pages for this purpose, due to its efficiency by using small hash fingerprints for comparison.

The above mentioned study by Adar et al. [8] used the Sørensen-Dice coefficient [25, 26] to compare the textual content of the same web resource at different times during their crawl.

Figuerola et al. [27] demonstrated that Kornblum’s Spamsum algorithm [28] can successfully be used to support web crawlers. By conducting tests on more than 80,000 web pages, they established a threshold score of 0.9 to determine if pages had changed between crawls. Jackson also used Spamsum in his 2015 analysis of the UK Web Archive’s holdings [20] mentioned above.

The Jaccard coefficient [29] was, for example, used by Sivakumar [30] in 2015. The purpose of the study was to ultimately improve search results by comparing blocks of text within web pages, as well as identifying and removing duplicate advertisements, headers, and other recurring features present in web sites. Other applications of the Jaccard coefficient include document clustering (Karun, Philip, and Lubna [31]) and keyword similarity analysis (Niwattankul et al. [32]).

Another very popular text similarity measure is cosine similarity [33, 34]. It was, for example, applied by Hajishirzi, Yih, and Kolcz [35] for the detection of duplicate web pages and by Sandhya and Govardhan [36] for comparing documents as part of their document clustering approach. For AlNoamany, Weigle, and Nelson [37] the use of the cosine similarity measure proved essential to detect off-topic web pages in various web archive collections from Archive-It.

In addition to the above mentioned five similarity measures, other approaches have been demonstrated in the past. Examples include computing the Levenshtein distance between two DOM trees [38, 39] and calculating the deltas in embedded resources [40] of web pages. However, if the application of a similarity measure involves the HTML elements of web pages, the process relies upon the fact that the structure of the resources to be compared has not been altered. Since web archives often inject additional HTML elements for user experience and branding, and hence alter the DOM structure of our Pre and Post Mementos, we can not use these measures of similarity. Also, many of our references are not in HTML format, but are instead PDF documents, which renders any DOM tree comparison inapplicable. Further methods to assess similarity rely upon a vast corpus to compute similarity [41] and others, such as Hamming distance [42], are suitable for same-length text strings only. Neither of these methods are suitable for our experiment since we seek methods that compare two documents without knowledge of a larger corpus and we can not rely on equal length of web pages.

## Methods

Our study uses the same URI references to web at large resources, 1,059,742 in total, that were used for [1]. We briefly describe how these URI references were obtained and refer to [1] for a detailed description. The URI references were extracted from three scholarly corpora consisting of 3.5 million articles published between 1997 to 2012: arXiv, Elsevier, and PubMed Central (PMC). We downloaded all articles from arXiv and PMC published in that time frame and used a (meanwhile discontinued) CrossRef API to randomly select DOIs of Elsevier articles. The arXiv corpus mainly covers physics, mathematics, and computer science; the Elsevier corpus covers a wide range of STM subjects; PMC mostly covers biomedical and life sciences. After processing these corpora, for example removing articles without URI references, a dataset consisting of 707,667 arXiv articles, 655,040 Elsevier articles, and 479,194 PMC articles remained. Next, all URIs were extracted from each article using a highly accurate regular expression-based URI extraction approach that is described in [43]. URIs were extracted from all sections of an article including the abstract, the body, footnotes, and the reference section. This resulted in a total of 3,983,985 URIs references, which were classified into three categories:

1. References to web at large resources
2. References to journal articles
3. References to be excluded, for example, because of URI syntax errors.

References to web at large resources were the focus of the research reported in [1] and are the starting point of the research reported here too. A total of 1,059,742 of such URIs were extracted from the three corpora. As shown in the top row of Table 1, 346,177 of those URIs are from arXiv papers, 232,712 from Elsevier papers, and 480,853 were extracted from PMC papers.

**Table 1:** Obtaining Pre/Post Mementos for URI References.

	arXiv	Elsevier	PMC	Total
URI references	346,177	232,712	480,853	1,059,742
No TimeMap	62,923	34,897	54,068	151,888
No Memento Pre	36,942	11,870	50,357	99,169
No Memento Post	17,027	22,303	23,140	62,470
Problem dereferencing Memento	18,594	18,525	28,960	66,079
**URI references with Pre/Post Mementos**	**210,691**	**145,117**	**324,328**	**680,136**

We use these URIs for two experiments. The first is aimed at assessing to what extent representative Mementos of URI references to web at large resources exist in web archives worldwide. The second is aimed at assessing the extent of content drift for URI references to web at large resources in scholarly papers. It uses only those URIs for which representative Mementos are found in the first experiment.

### Web Archive Lookup of URI References

Web archives continuously create snapshots of URIs around the web. Naturally, they may also take snapshots of URIs that are referenced in scholarly papers. For each URI for which snapshots exist, the Memento protocol [19] and its associated aggregation infrastructure provide a TimeMap that gives an overview of all Mementos for the URI held by public web archives worldwide. Each TimeMap lists the original URI as well as the URI and the archival date of each of its Mementos. At the time the experiment was conducted, the aggregation infrastructure covered all existing web archives that exposed an openly accessible machine interface. There were 19 in total, only 6 of which were also used in [1]: Archive-It, archive.is, Bibliotheca Alexandrina Web Archive, Canadian Government Web Archive, Croatian Web Archive, Estonian Web Archive, Icelandic Web Archive, Internet Archive, Library of Congress Web Archive, NARA Web Archive, Portuguese Web Archive, PRONI Web Archive, Slovenian Web Archive, Stanford Web Archive, UK National Archives Web Archive, UK Parliament Web Archive, UK Web Archive, Web Archive Singapore, and WebCite.

We programmatically obtain TimeMaps for all referenced URIs, and from each select two Mementos: the Memento Pre, which has an archival date that is temporally closest and prior to the publication date of the article that references the URI, and the Memento Post, which is temporally closest and past that date. This selection is motivated by the insight that, if these two Mementos are highly similar—the resource has hardly changed in the pre/post interval that surrounds the publication date—then these two Mementos are very likely representative of the referenced content as it was when the article was published. After having selected these two Mementos per URI reference, we obtain their archived content from the web archive they reside in by dereferencing the URI of the respective Memento found in the TimeMap.

This entire process was conducted over the course of August 2015. To address possible temporary glitches, the process was repeated once for those URIs for which the process had failed in the first run. Table 1 shows the outcome. As can be seen, we were able to obtain content for Pre/Post Mementos for 680,136 of the 1,059,742 URI references in our collection. The Table also details the reasons why the process was not successful for the remaining 379,606 URIs. These include the unavailability of TimeMaps (no Mementos exist), the unavailability of either Pre or Post Mementos (Mementos exist only pre or only post the publication date), and several instances whereby dereferencing a Memento’s URI failed. For URI dereferencing, we used the command line tool cURL to issue HTTP GET requests and configured cURL to follow a generous maximum of 50 HTTP redirects. Conducting this process was straightforward for all web archives except WebCite, an archive that was explicitly introduced more than a decade ago to combat reference rot in scholarly communication. This archive sets a very low and seemingly variable limit to the number of requests a client can issue on a daily basis. Also, programmatically obtaining Mementos from this archive required extracting content from frames; therefore we used the PhantomJS [44] browser automation tool instead of cURL [45].

### Assessing the Representativeness of Mementos

We use the 680,136 URI references that remain from the process described above. For each of these references, a Pre and Post Memento exists. Our goal is to measure the similarity between these Mementos and then to declare highly similar ones as representative of the referenced content as it existed at the time of publication of the referencing paper. Doing so involves two distinct challenges: measuring similarity and setting a threshold for a similarity measure above which content is regarded as sufficiently similar.

#### Measuring Similarity

An inspection of the content types of our Pre and Post Mementos, summarized in Table 2, shows that the vast majority of Mementos are textual. Over 1 million are text/html, about 93,000 are application/pdf, and about 5,000 are text/plain. The numbers for other content types are very small in comparison. Since well established techniques exist for measuring the similarity of textual content, we decide to only retain Mementos with these three most frequently occurring content types. This leads to dismissing 10,112 Pre/Post Mementos, which corresponds to 5,056 URI references.

**Table 2:** Top 10 Content Types of Pre and Post Mementos.

Content Type	Mementos	In%
text/html	1,041,192	90.68%
application/pdf	93,428	8.14%
text/plain	5,424	0.47%
application/postscript	1,690	0.15%
application/msword	911	0.07%
application/vnd.ms-excel	610	0.05%
image/gif	493	0.04%
application/xhtml+xml	484	0.04%
application/octent-stream	447	0.04%
image/jpeg	405	0.04%

In order to be able to compare the content of the remaining Mementos, we extract the text from the HTML and PDF files. This obviously involves removing tags from the HTML and control characters from the PDF. We respectively use lxml [46] and pyPDF [47] to achieve this. However, the text extraction process posed significant additional challenges that required writing custom code. Detailing these is beyond the scope of this paper but a technical report on the matter is available [45]. For the purpose of this discussion it suffices to mention that the most common challenge was removal of content inserted into Mementos by web archives, including JavaScript, CSS, and textual information. Table 3 provides an overview of URI references with Pre/Post Mementos that were excluded from the textual comparison because their content type was not one of the top three selected types or because the text extraction process was unsuccessful. We are left with 648,253 URI references for which the textual content of Pre/Post Mementos can be compared.

**Table 3:** Selecting Pre/Post Mementos for Textual Comparison.

	arXiv	Elsevier	PMC	Total
URI references with Pre/Post Mementos	210,691	145,117	324,328	680,136
Not in top 3 content types	1,358	1,296	2,402	5,056
Text extraction processing errors	1,562	3,064	13,669	18,295
No content after text extraction	2,864	1,720	3,948	8,532
**URI references for Pre/Post Memento comparison**	**204,907**	**139,037**	**304,309**	**648,253**

With the extracted text from our Pre/Post Memento pairs remaining, we proceeded to assess their similarity. We do so by using the text similarity measures introduced in the Related Work section, and their corresponding implementations, shown in Table 4. These four measures were selected because their specific and complementary characteristics provide insight into different notions of similarity. Hence, their combination offers a well-rounded view of changing textual content.

**Table 4:** Text Similarity Measures, Implementations, and Score Ranges before Normalizing.

Similarity Measure	Implementation	Maximum Similarity Score	Minimum Similarity Score
Simhash	Simhash Python module [50]	0	64
Jaccard	Python distance module [51]	0	1
Sørensen-Dice	Python distance module [51]	0	1
Cosine	Python scikit-learn module [52]	1	0

We use Simhash, a hash-based measure, that splits two strings into n-grams and creates one vector of n-grams per string. It then computes hash values for both vectors and returns their distance indicating the similarity of both strings. Simhash is often used for large-scale web page comparisons and is designed to be sensitive to editorial changes in the compared texts. We initially also used Spamsum as a byte-level similarity measure since it was applied in previous related work [20]. However, we found many instances in which the Spamsum score differed dramatically from other scores and found the explanation in [48, 49] where the creator of the algorithm states that input strings need to be more than 4KB of length for the measure to provide meaningful results. Since this was not the case for about 70% of our comparisons, we decided to dismiss Spamsum. Our second and third methods are the Jaccard and the Sørensen-Dice coefficients, two common low-level string similarity metrics. Compared to Simhash, both are more forgiving when it comes to minor editorial changes in the text as they only consider the sets of shared unique characters. We therefore expect them to provide a different indicator of text similarity or diversity. Since none of these three selected measures provide insights into the contextual changes of the compared documents, we also include cosine similarity. This measure is designed to indicate the degree to which the semantic content of compared texts differ, based on changes to salient terms.

Since the implementations of the selected measures have different ranges to express similarity, we normalize the respective scores according to Eqs (1) through (4). The normalized scores all have a range between 0 and 100, with 0 indicating least similar and 100 most similar.

$$\begin{array}{c} Simhash_{n}\left(d_{1},d_{2}\right)=100(1-\frac{Simhash(d_{1},d_{2})}{64}) \end{array}$$ (1)

$$\begin{array}{c} Jaccard_{n}\left(d_{1},d_{2}\right)=100\left(1-Jaccard\left(d_{1},d_{2}\right)\right) \end{array}$$ (2)

$$\begin{array}{c} SørensenDice_{n}\left(d_{1},d_{2}\right)=100\left(1-SørensenDice\left(d_{1},d_{2}\right)\right) \end{array}$$ (3)

$$\begin{array}{c} Cosine_{n}\left(d_{1},d_{2}\right)=100\left(Cosine\left(d_{1},d_{2}\right)\right) \end{array}$$ (4)

To better understand the differences between the four text similarity measures, consider the following string comparison example of the strings *s*_1_ = “*birds eat cats*”, *s*_2_ = “*cats eat birds*”, and *s*_3_ = “*birds eat cake*”. *s*_1_ and *s*_2_ contain the same terms, but the order is different, and *s*_1_ and *s*_3_ are identical except for the last term. Results of the application of our four methods to compare strings *s*_1_ with *s*_2_ and *s*_1_ with *s*_3_, respectively, are summarized in Table 5. For the comparison of *s*_1_ with *s*_2_ the Simhash value is, as expected, below 100 because the changed order of terms affects the hashes that are compared. Jaccard and Sørensen-Dice, on the other hand, are set-based and hence their score is not affected by the changed order of terms. Cosine also returns a perfect score because all salient terms of *s*_1_ ocurr also in *s*_2_. The picture changes for the comparison of *s*_1_ with *s*_3_. The Simhash value is sightly higher than before because *s*_1_ and *s*_3_ only differ by two characters. The values for Jaccard and Sørensen-Dice are not perfect anymore since the strings differ by one term. Compared to Jaccard, however, Sørensen-Dice is slightly more forgiving for the same changes. It is important to note that the Cosine score drops as one salient term in *s*_1_ has been replaced with another in *s*_3_.

**Table 5:** Example String Comparison Using the Four Similarity Measures.

*s*_1_ = “*birds eat cats*” *s*_2_ = “*cats eat birds*” *s*_3_ = “*birds eat cake*”		
	*s*_1_ vs. *s*_2_	*s*_1_ vs. *s*_3_
Simhash	67.2	73.4
Jaccard	100.0	90.9
Sørensen-Dice	100.0	95.2
Cosine	100.0	50.3

#### Establishing a Similarity Threshold

Since the main goal of our research is to assess the extent of content drift for URI references in scholarly articles, we want to ensure that we compare Mementos that are truly representative of referenced URIs with the content of those URIs on the live web. Therefore, we use a stringent similarity threshold and consider a Pre/Post Memento pair similar, and hence representative, only if the Mementos have the maximum score of 100 for all similarity measures.

We assess the appropriateness of this threshold by considering a subset of Pre/Post Memento pairs that are considered the same according to the HTTP protocol, from here on referred to as **HTTP-same**. HTTP has two response headers—Etag and Last-Modified—that are used to indicate whether a resource’s representation has changed: if two representations of a resource with a given HTTP URI have the same value for the Etag or Last-Modified header, then these representations are the same [53]. Some web archives preserve the original Etag and Last-Modified headers, if they were present in the resources for which a Memento was created. Specifically, web archives that operate the Wayback software convey them as X-Archive-Etag and X-Archive-Last-Modified headers for Mementos [54]. Hence, we select from our collection of Pre/Post Mementos the subset for which either the X-Archive-Etag or the X-Archive-Last-Modified response header exists and has the same value for the Pre and the Post Memento. We find such Memento pairs for 120,734 of the remaining total of 648,253 URI references.

**Fig 5:**
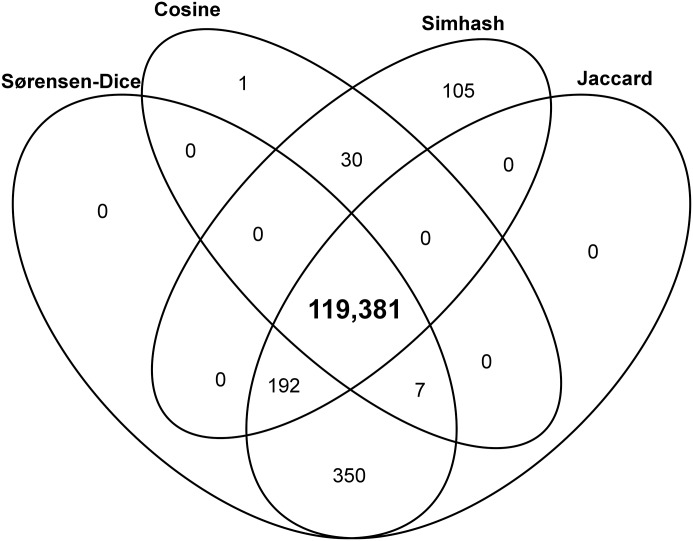
Pre/Post Memento Pairs, which are HTTP-same, that have Maximum Similarity for One or More Measure.

According to HTTP, the selected pairs of Pre and Post Mementos are the same. We proceed by computing their similarity according to the normalized measures introduced above. Fig 5 only considers Pre/Post Mementos that have the maximum similarity score according to one or more measures. At the center, we find 119,381 out of 120,734 (98.88% of our subset) that have the maximum similarity score for all measures. We also find a limited number of cases where not all similarity measures agree, that is, where certain measures do not yield the maximum score when comparing Mementos that are HTTP-same. Table 6 shows the percentage of the selected Pre/Post Memento pairs that have a maximum similarity score for each considered measure. We find that, in the vast majority of cases (98.91% and higher), Pre/Post Memento pairs that are the same according to HTTP also have the maximum similarity score according to one of the measures. The percentage of pairs for which this is not the case (1.09% and lower) fully aligns with percentages found in previous work that exposed unreliable implementations of the Etag or Last-Modified headers [55]. They are cases for which a server claims the representations are the same while they are not. These observations give us confidence that requiring a maximum similarity score for all measures will yield Pre/Post Memento pairs that are truly representative.

**Table 6:** Percentages of Pre/Post Memento Pairs, which are HTTP-same, that have Maximum Similarity per Measures.

Similarity Measure	Percentage of HTTP-same Pre/Post Memento Pairs with Maximal Textual Similarity
Simhash	99.15%
Jaccard	99.33%
Sørensen-Dice	99.33%
Cosine	98.91%

#### Selecting Representative Mementos

**Fig 6:**
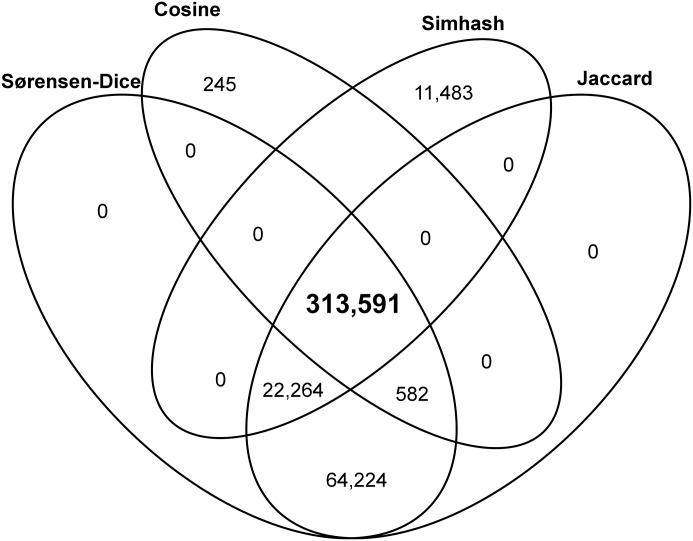
Pre/Post Memento Pairs with Maximum Similarity for One or More Measure.

We apply our stringent similarity threshold to the collection of 648,253 URI references for which Pre/Post Memento pairs can be compared as per Table 3, and find 313,591 (48.37%) for which the Pre/Post Memento pairs have the maximum similarity score for all measures; these Mementos are considered representative. Table 7 provides the numbers per corpus in the top row. Fig 6 shows only URI references with Pre/Post Mementos that have a maximum similarity score for one or more measures. The URI references with representative Mementos are at the center of the figure.

**Table 7:** Obtaining Live Web Content for URI References with Representative Mementos.

	arXiv	Elsevier	PMC	Total
URI references with representative Mementos	102,069	73,949	137,573	313,591
No live web content	19,321	20,883	26,867	67,071
Content type drift	338	863	2,105	3,306
Text extraction processing errors	42	55	125	222
No content after extraction	770	391	740	1901
**URI references for content drift assessment**	**81,598**	**51,757**	**107,736**	**241,091**

It needs to be noted that we chose the stringent similarity threshold in order to select a set of Mementos that very precisely reflects the state of referenced resources as they were when the referencing paper was published. Establishing the ground truth in this manner is essential in order to be able to accurately assess the extent of content drift by comparing the selected Mementos with their counterparts on the live web. Using a more lenient threshold would decrease the confidence that Mementos truly reflect that past state, but would yield a higher count of representative Mementos. For example using a threshold that requires a maximum score for only one of the similarity measures results in 413,918 (63.9% out of 648,253) Mementos that might be representative. But as Table 5 shows, under such a threshold *s*_1_ and *s*_2_ would be selected as representative Pre/Post Memento pairs.

### Live Web Lookup of URI References with Representative Mementos

Since we have now selected 313,591 URI references for which representative Mementos are available in web archives around the world, we can proceed to assess to what extent the content of these references has drifted since the publication of the referencing paper. We do so by dereferencing each of these URI references on the live web. This was done over the course of August of 2015. We used the command line tool cURL to issue HTTP GET requests and, again, allowed cURL to follow a maximum of 50 HTTP redirects. The process was repeated once for those URIs that dereferenced unsuccessfully in the first pass. As is to be expected, we were unable to obtain live web content for a significant number of URI references (67,071 out of 313,591, which is 21.4%). We further limit the number of references by removing those for which the content type has drifted away from the top three content types we considered for Mementos as well as those for which the text extraction process is unsuccessful or yields no content at all. Table 7 provides an overview of the described actions, which lead to retaining 241,091 URI references for which we can reliably assess content drift.

### Assessing Content Drift between Representative Mementos and Associated Live Web Representations

**Fig 7:**
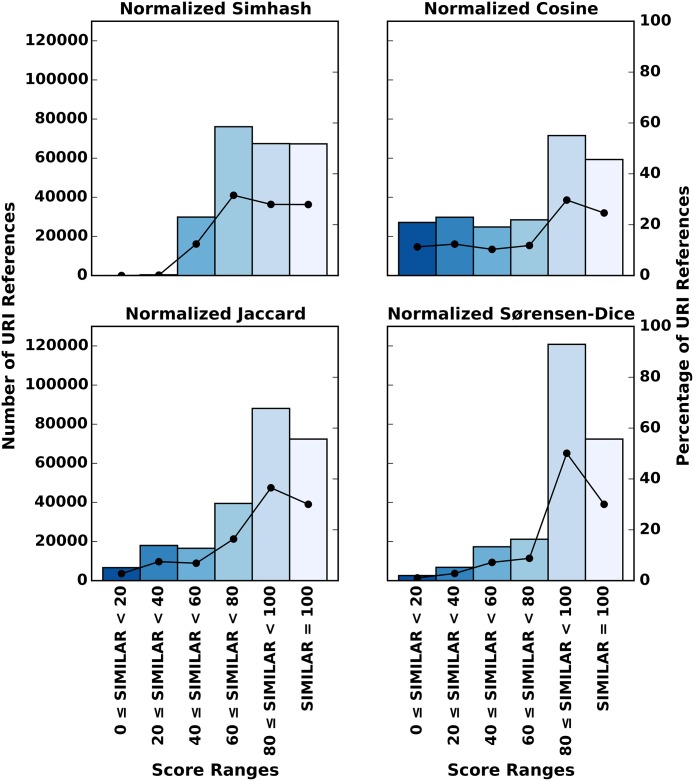
Histograms and Ratios per Normalized Measure for Similarity Scores of Comparing Representative Mementos and Live Web Content—All Corpora.

The respective Pre/Post Memento pairs for the considered 241,091 URI references have the maximum similarity score for all normalized measures. For each pair, we select the Memento with an archival date closest to the publication date of the paper in which the URI reference occurs and compare it to that URI’s live web counterpart using each of the normalized similarity measures. As noted above, each of our measures is known to have its specific characteristics and to respond differently to textual changes in compared texts. Fig 7 visualizes the differences with a histogram of the similarity scores for each measure that result from comparing the selected Memento and the live web resource. The absolute numbers are reported with bars that correspond to the left y-axis and their relative counterparts are indicated with lines that correspond to the right y-axis. We can identify a clear pattern: the vast majority of similarity values fall into the right half of the histograms, representing normalized similarity values above 60. Between 70% and 80% of the Jaccard and Sørensen-Dice coefficients even fall into the top two categories with values above 80. Cosine values are more evenly distributed and so are Simhash values. Note that, due to the implementation of the Simhash algorithm that computes the distance between documents as the number of bytes out of a maximum of 64, its normalized value only infrequently drops below 40.

In order to provide a concise insight into content drift for our remaining referenced URIs, we introduce a combined measure, the **aggregate similarity indicator**, which is the mean of normalized scores for all similarity measures. Table 8 illustrates the computation of this indicator for the comparisons of the three example strings *s*_1_, *s*_2_, and *s*_3_ introduced in Table 5.

**Table 8:** Computation of the Aggregate Similarity Indicator.

Measure	*s*_1_ vs. *s*_2_	*s*_1_ vs. *s*_3_
Simhash	67.2	73.4
Jaccard	100	90.0
Sørensen-Dice	100	95.2
Cosine	100	50.3
**Aggregate Similarity Indicator**	**91.8**	**77.5**

Even though our four similarity measures provide indicators for different aspects of textual similarity, the distribution of absolute as well as relative scores, as displayed in Fig 7, shows that all measures return values that fit a same pattern. Therefore, we are encouraged to utilize the aggregate score as an appropriate numeric representation of the similarity of two compared textual documents. For completeness, we provide the results of the individual measures in the Supporting Information section.

## Results

### Representative Mementos

As mentioned previously, we find that for about 30% of URI references (313,591 out of 1,059,742), Mementos that are verifiably representative exist. For those references, the past context can reliably be revisited. The remaining 70% of references belong to different categories (Tables 1, 3 and 7). For some (14.33%), the past context can definitely not be revisited because there are no Mementos. For others (15.25%), we can not verify whether Mementos are representative, because no Pre or Post Memento exists. For yet others (8.77%), the existing Mementos were problematic either because they were inaccessible in the archive that claimed they existed or because processing them was unsuccessful, both likely a sign of corrupt archival content. For the remainder of URI references (31.58%), Pre/Post Mementos exist but their similarity was below the threshold we set, supported by insights gained from textually comparing HTTP-same Mementos.

**Fig 8:**
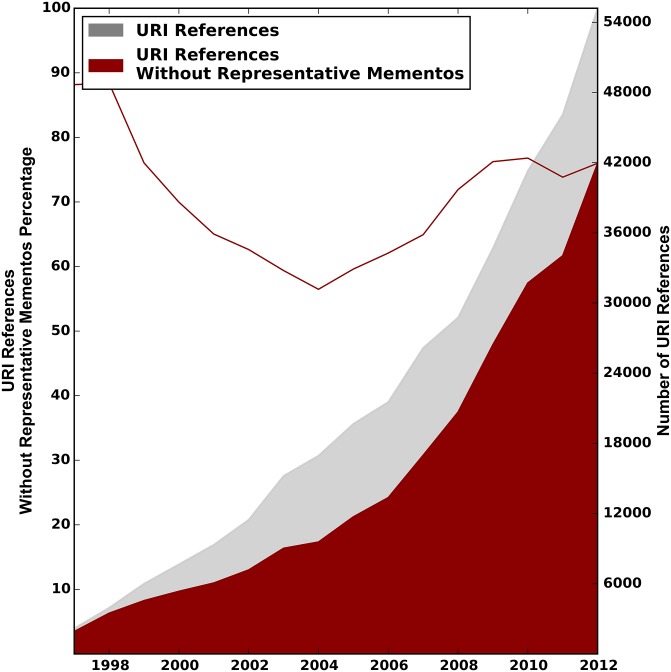
URI References without Representative Mementos—arXiv Corpus.

**Fig 9:**
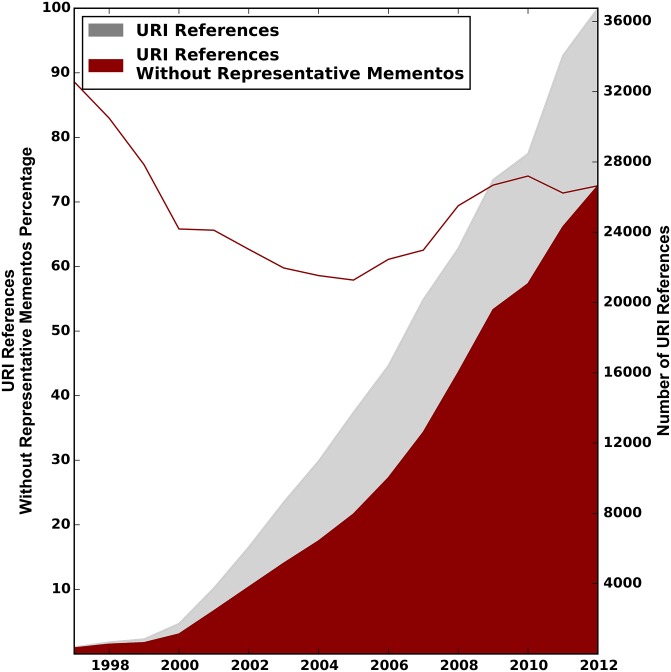
URI References without Representative Mementos—Elsevier Corpus.

**Fig 10:**
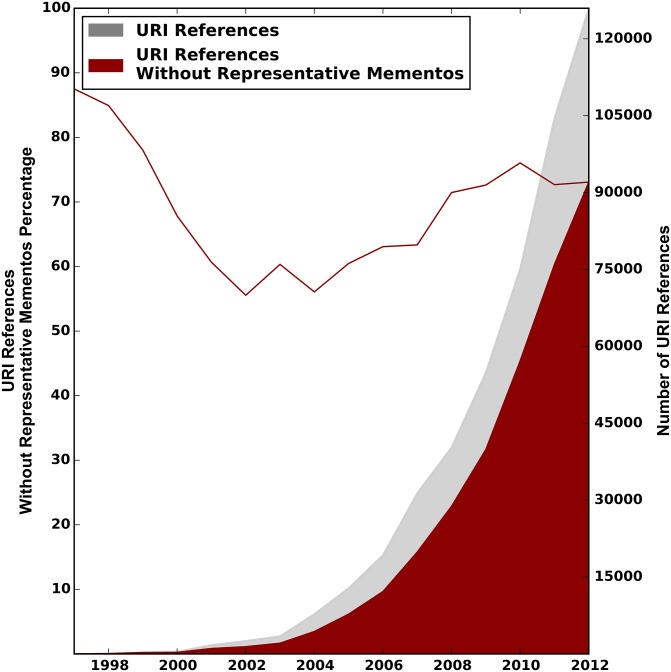
URI References without Representative Mementos—PMC Corpus.

Figs 8–10 show the distribution of URI references that lack representative Mementos per publication year of the referencing paper, for the arXiv, Elsevier, and PMC corpus, respectively. These are the URI references for which the past context can not be revisited. The gray surface of the plots depicts the total number of web at large references, as per the first row of Table 1. The overlaid red surface shows the total number of URI references without representative Mementos, which is the total number of URI references minus those with representative Mementos as per the first row of Table 7. The right hand y-axis shows the absolute numbers. The solid red line in the same figures, to be interpreted using the left y-axis, shows the percentage of URI references that lack representative Mementos per publication year of the referencing article.

For all corpora, we see that the total number of references without representative Mementos grows over time at about the same pace as the number of URI references to web at large resources. Percentage wise, the curve starts around 88% of references without representative Mementos in 1997, and then reaches a minimum of about 58% around 2004, meaning representative Mementos exist for about 42% of URI references in that publication year. But this trend does not continue past 2004, and by 2012 we find about 70% of references without representative Mementos. The evolution across publication years is surprisingly similar for all corpora. Also, the downward trend for more recent publication years is rather surprising because the Internet Archive, which holds most of our Mementos, has adopted a more aggressive crawl strategy in recent years, and more archives have come online. Possible explanations for this trend include the growth rate for references to web at large resources exceeding the growth in global web archiving activity, a change rate of referenced resources that is faster than the re-crawl frequency of archives, and a collection policy by web archives that somehow bypasses URIs that are referenced in scholarly communication.

### Content Drift

**Fig 11:**
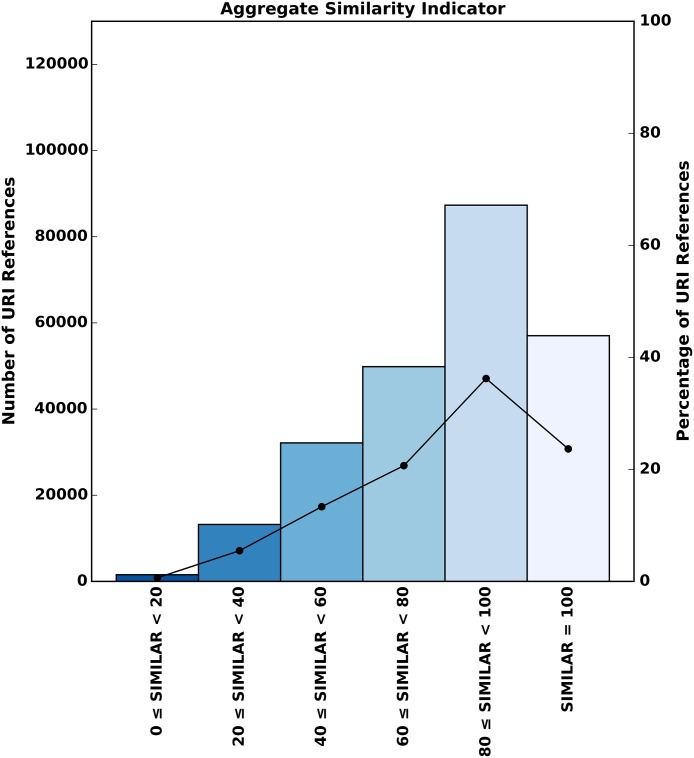
Distribution of Absolute and Relative Aggregate Similarity Indicator Scores.

We assess the extent of content drift as it happened between the time of publication of the referencing article and the current, consultation time. To that end, we consider the 241,091 URI references for which we found representative Pre/Post Memento pairs as well as corresponding live web content, as per Table 7. We compute each of the similarity measures comparing the representative Memento and the live resource. Fig 11 shows the distribution of absolute and relative aggregate similarity scores across all corpora and as such represents an aggregation of the four histograms shown in Fig 7. We find a total of 57,026 (23.65%) URI references that have not been subject to content drift. In other words, the content on the live web has drifted away from the content that was originally referenced for three out of four references (184,065 out of 241,091, which equals 76.35%).

**Fig 12:**
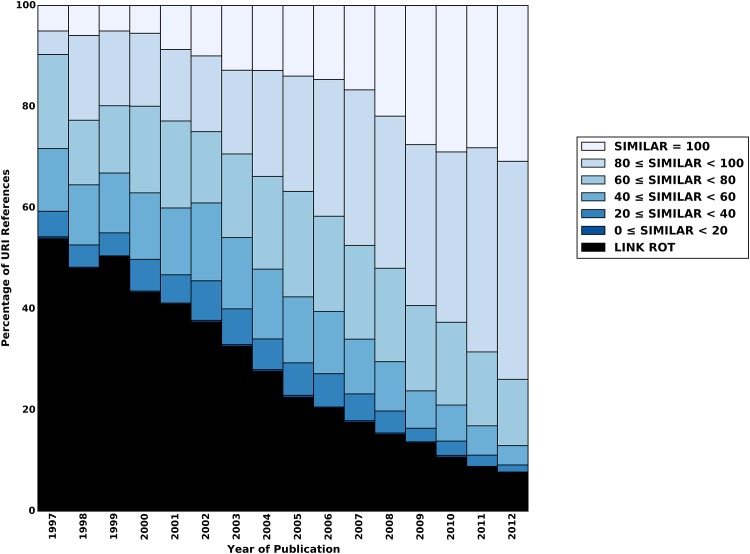
Similarity Ranges for Representative Mementos and Live Web Content Per Publication Year—arXiv corpus.

**Fig 13:**
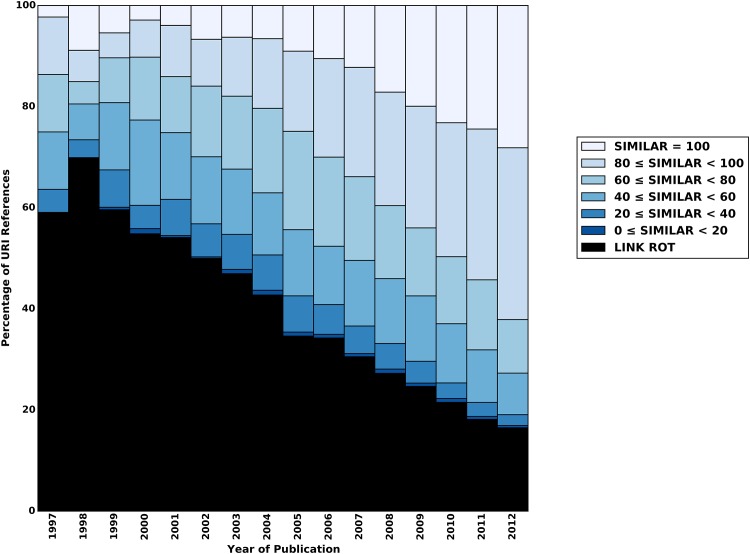
Similarity Ranges for Representative Mementos and Live Web Content Per Publication Year—Elsevier corpus.

**Fig 14:**
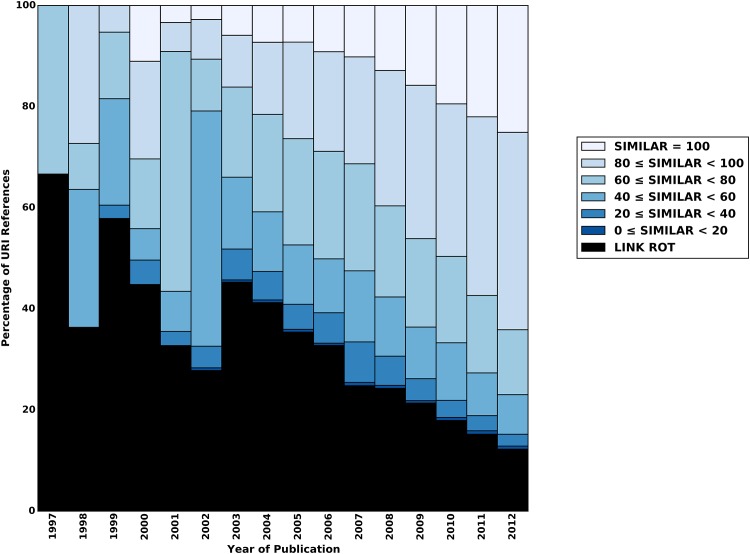
Similarity Ranges for Representative Mementos and Live Web Content Per Publication Year—PMC corpus.

For a more detailed analysis of content drift, we also evaluate the results per corpus and per publication year of the referencing articles. Figs 12–14 show scores of the aggregate similarity indicator for arXiv, Elsevier, and PMC, respectively. The various colors in these figures correspond with ranges of the aggregate similarity indicator. The lightest blue color indicates the maximal similarity score. As similarity decreases, the blue gets darker. URI references that are subject to link rot are shown in black. The publication year of the referencing article is shown on the x-axis, and the percentage of URI references that belong to a category (e.g. a similarity range, link rot) is shown on the y-axis. The URI references that have not been subject to content drift are shown in the lightest blue at the top of the bars.

We see for all corpora that even for articles published in 2012 only about 25% of referenced resources remain unchanged by August of 2015. This percentage steadily decreases with earlier publication years, although the decline is markedly slower for arXiv for recent publication years. It reaches about 10% for 2003 through 2005, for arXiv, and even below that for both Elsevier and PMC. For 1997, admittedly a publication year for which very few URI references and Mementos exist, hardly any unchanged resources exist for arXiv and Elsevier. For PMC, none exist at all for 1997 through 1999. When being lenient by including all similarity scores above 80 as “similar enough”, we find that, since they were referenced in 2012, about 60% of resources have somehow remained stable for Elsevier and PMC, and about 70% for arXiv. That percentage drops to about 40% for 2009 in the case of Elsevier and PMC, while arXiv does better with 60%. For 1997, arXiv and Elsevier have about 10% of references in this category, and PMC has none. arXiv does better than the other corpora both regarding maximum similarity and the more lenient above 80 score. Overall, arXiv and Elsevier show a relatively uniform and steady pattern. PMC has a similar pattern for more recent publication years but a more erratic one for the early publication years. Most likely, this is related to the following observation made in [1]: “the PMC corpus is very small for early publication years but it picks up, initially slowly around 2005, and then rather abruptly around 2007. This growth pattern is related to the PMC submission policy that changed from voluntary to mandatory in 2008.” Regardless, all three figures clearly show that the discrepancy between the live content and representative Mementos grows over time, thereby providing strong evidence that content drift significantly affects URI references made in scholarly articles.

For the sake of completeness and comparison we provide a plot per corpus and per similarity measure in the Supporting Information section. These plots have the same layout as Figs 12–14 and overall display the same trends. Note that the cosine similarity measure reveals the same trend, indicating that the observed content drift is not merely the result of character changes but also caused by semantic drift of referenced URIs over time.

## Discussion

Our results indicate that the extent of content drift for references to web at large resources in the STM literature is very significant. It is conceivable that the impact of content drift may be less important for some URI references than for others. For example, one could argue that content drift is less detrimental for a reference to a project web site than for a reference to specific content. Proving this intuition would require surveying authors about the intentionality of their references and readers about their perception of the appropriateness of referenced content some time after the references were made. It would also have to involve automatically characterizing URIs as referring to, for example, a project, organization, or a tool versus specific content. Such an exploration is beyond the scope of this work, which focuses purely on verifiably quantifying the extent of content drift. We do offer an initial insight into this orthogonal problem domain by providing two low-level characterizations of the web at large URIs referenced in our corpora. Table 9 shows the distribution of URI depth, calculated by counting the number of components of a URI’s path and adding 1 if a query string is encountered. The data shows that the most frequently referenced URIs are rather short but that over two thirds of URIs have a depth greater than or equal to 1. Table 10 lists the 25 most frequently referenced URIs. It reveals that URIs that reference projects, etc. can have a path depth greater than zero. These observations indicate that automatically classifying URIs along the aforementioned dimensions would be challenging.

**Table 9:** Distribution of References by URI Depth.

Depth	References	Depth	References
0	340,498	10	294
1	177,016	11	120
2	222,157	12	96
3	161,120	13	23
4	87,281	14	17
5	42,467	15	13
6	16,494	16	4
7	9,327	17	2
8	2,059	21	1
9	752	28	1

**Table 10:** 25 Most Frequent URI References.

Frequency	URI
6,697	http://www.r-project.org/
3,470	http://www.imstat.org/
2,666	http://www.sdss.org/
1,944	http://genome.ucsc.edu/
1,690	http://www.rupress.org/terms
1,612	http://rsb.info.nih.gov/ij/
1,408	http://pdg.lbl.gov/
1,365	http://www.pymol.org/
1,326	http://www.ensembl.org/
1,215	http://www.geneontology.org/
1,182	http://www.clinicaltrials.gov/
1,061	http://clinicaltrials.gov/
1,050	http://www.imstat.org/aos
1,025	http://www.ingenuity.com/
998	http://www.bioconductor.org/
968	http://www.ccdc.cam.ac.uk/
913	http://blast.ncbi.nlm.nih.gov/Blast.cgi
877	http://www.ccdc.cam.ac.uk/conts/retrieving.html
873	http://www.slac.stanford.edu/xorg/hfag
807	http://www.icmje.org/
805	http://rsbweb.nih.gov/ij/
799	http://www.arabidopsis.org/
786	http://www.ccdc.cam.ac.uk/data_request/cif
748	http://www.hapmap.org/
738	http://www.repeatmasker.org/

Table 11 provides a categorization of URI references using two dimensions: whether a representative Memento exists for the reference and whether live web content is available for it. The numbers in the table show where the 1,059,742 URI references used for this study belong in this categorization. The table also shows the URI references for which processing errors were encountered, as per Tables 1, 3 and 7. The numbers are alarming in their own right as they show the lack of representative Mementos for the majority of URI references, highlight the remarkable extent of content drift, and confirm the severity of link rot found in many previous studies, including [1], which was based on this very set of URI references. Making the unverifiable assumption that the same content drift ratio (76.35% as seen above) holds for URI references with and without a representative Memento, we can deduce that for 533,785 out of 699,130 references content has drifted since they were originally referenced. In addition, 257,221 references suffer from link rot. Discounting the 103,391 URI references for which processing errors were encountered, that makes a total of 791,006 URI references out of 956,351 (82.71% or about four out of five) that suffer from reference rot, the combination of link rot and content drift. Note also the 190,150 URI references (18% of the URI collection) for which neither live web content nor a representative Memento is available. Any trace of these references has vanished.

**Table 11:** A Categorization of the 1,059,742 URI References in Terms of the Existence of Representative Mementos and Live Web Content.

	Representative Memento	No Representative Memento	Total
**Live web content**	241,091	458,039	**699,130**
Not drifted	57,026	n/a	n/a
Drifted	184,065	n/a	n/a
**No live web content**	67,071	190,150	**257,221**
**Error**	5,429	97,962	**103,391**
**Total**	**313,591**	**746,151**	**1,059,742**

In order to put our work into perspective and highlight the significance of our findings, we compare the results with related work. In particular, we found representative Mementos for about 30% of URI references (313,591 out of 1,059,742) compared to around 25% shown in Klein et al. [1]. However, our Mementos are verifiably representative whereas in [1] a Memento was declared representative if it was created within a 14-day window around the referencing article’s publication date. In addition, our analysis results in a content drift ratio of around 76% compared to around 50% shown in the study conducted by Zittrain et al. [21] on US Supreme Court opinions. Note, however, that their evaluation of content drift was done manually and lacked a ground truth of representative Mementos. Hence, it represents a very lenient method to assess content drift compared to ours, which is based on a stringent selection of representative Mementos and the use of various text similarity measures. Zittrain et al. also found that between 65% and 73% of URI references from three law journals suffer from either link rot or content drift. Given the numbers presented in Table 11 we can conclude that 21.8% of our URI references with representative Mementos suffer from link rot (67,071 out of 308,162) and 59.7% (184,065 out of 308,162) suffer from content drift. The sum of 81.5% represents the number of URI references from our vast and diverse corpus of scholarly articles that suffer either from link rot or content drift. These comparisons show that our results are similar to those found in related studies. However, our numbers are verifiable and do not rely on estimations or manual interpretations of content drift.

Beyond the mere numbers, interesting considerations apply to these categories when approached from the perspective of web-based scholarly communication. When it comes to the URI references for which live web content is available, our findings regarding the extent of content drift raise the concern that a reader who follows a link to the live web is led to believe that the content at the end of that link is representative of what was originally referenced, where in reality it very well may not be. Ironically enough, no such misinterpretation is possible in cases where no live web content exists because the HTTP error message that the reader receives is unambiguous; there is no room to misinterpret provided information. In both cases, a seamless way to navigate from a URI reference to web archives that hold Mementos for the referenced URI would be helpful. This could be achieved by leveraging the Memento Time Travel infrastructure [56] to automatically look up Mementos for a URI reference with an archival datetime that is temporally closest to the publication date of the referencing paper. As shown in Table 1, this would actually yield a Memento in a vast majority of cases. However, given the lack of representative Mementos for URI references in the body of scholarly literature published thus far, there would be no guarantees that the selected Memento would be representative. It would be a best effort solution.

Going forward, however, link rot and content drift can be combatted. The Hiberlink [2] project concluded that two components are necessary to arrive at a solution:

- Pro-actively creating snapshots in web archives of web at large resources that are referenced in scholarly communication—This component is aimed at maximizing the number of URI references for which representative Mementos exist. Special-purpose archives such as perma.cc [21] and weblock.io [57] are stepping into this problem domain and are exploring avenues to meet the challenge of operating web archives for the long term.
- Appropriately referencing these snapshots in scholarly literature—This component is aimed at allowing seamless navigation from a URI reference to live web content and a representative Memento in a manner that provides appropriate guarantees that the reference remains robust over time. With this regard, the Hiberlink project proposed the Link Decoration [58] and Robust Link [59] approaches, which are demonstrated in [60].

These insights from the Hiberlink project along with the aforementioned concrete progress regarding necessary infrastructure have the potential to turn reference rot in scholarly communication into a thing of the past. However, embedding capabilities that leverage this infrastructure in existing authoring, reviewing, and publication workflows is a major challenge. Establishing a solution that can be used and accepted across the scholarly landscape will require significant effort and leadership. The nature and extent of the challenge bear some similarities to efforts that started in the nineties to combat link rot for references to scholarly papers. Digital Object Identifiers and associated infrastructure were introduced as a solution that became accepted across the scholarly landscape. Today, it provides a significant level of interoperability that ties distributed scholarly venues together. But reference rot for links to web at large resources is a different problem altogether. Indeed, the DOI solution fully relies on the custodians of DOI-identified resources to keep links to their content working over time. These custodians have strong incentives to invest in achieving link stability because usage of their collections is at stake. Custodians of web at large resources typically do not have such incentives. They are not academic publishers but rather administrators of web sites that happen to be linked to from a scholarly paper. They are typically not overly concerned about the longevity of their own web site, let alone of that of the web-based scholarly record. Reference rot is a problem with roots largely outside the scholarly communication community but that will have to be solved by that very community.

## Supporting Information

S1 Fig Similarity Ranges for Representative Mementos and Live Web Content Per Publication Year—arXiv corpus—Using Normalized Simhash. (TIF)

S2 Fig Similarity Ranges for Representative Mementos and Live Web Content Per Publication Year—arXiv corpus—Using Normalized Jaccard. (TIF)

S3 Fig Similarity Ranges for Representative Mementos and Live Web Content Per Publication Year—arXiv corpus—Using Normalized Sørensen-Dice. (TIF)

S4 Fig Similarity Ranges for Representative Mementos and Live Web Content Per Publication Year—arXiv corpus—Using Normalized Cosine. (TIF)

S5 Fig Similarity Ranges for Representative Mementos and Live Web Content Per Publication Year—Elsevier corpus—Using Normalized Simhash. (TIF)

S6 Fig Similarity Ranges for Representative Mementos and Live Web Content Per Publication Year—Elsevier corpus—Using Normalized Jaccard. (TIF)

S7 Fig Similarity Ranges for Representative Mementos and Live Web Content Per Publication Year—Elsevier corpus—Using Normalized Sørensen-Dice. (TIF)

S8 Fig Similarity Ranges for Representative Mementos and Live Web Content Per Publication Year—Elsevier corpus—Using Normalized Cosine. (TIF)

S9 Fig Similarity Ranges for Representative Mementos and Live Web Content Per Publication Year—PMC corpus—Using Normalized Simhash. (TIF)

S10 Fig Similarity Ranges for Representative Mementos and Live Web Content Per Publication Year—PMC corpus—Using Normalized Jaccard. (TIF)

S11 Fig Similarity Ranges for Representative Mementos and Live Web Content Per Publication Year—PMC corpus—Using Normalized Sørensen-Dice. (TIF)

S12 Fig Similarity Ranges for Representative Mementos and Live Web Content Per Publication Year—PMC corpus—Using Normalized Cosine. (TIF)
