# Correction: Scholarly Context Adrift: Three out of Four URI References Lead to Changed Content

**DOI:** 10.1371/journal.pone.0171057

**Published:** 2017-01-25

**Authors:** The PLOS ONE Staff

There is an error in affiliation 2 for authors Richard Tobin and Claire Grover. Affiliation 2 should be: Language Technology Group, The University of Edinburgh, Edinburgh, Scotland, United Kingdom. The publisher apologizes for the error.
