# Supplementary figures and images for: Scholarly Context Adrift: Three out of Four URI References Lead to Changed Content
Source: PLoS One. 2016 Dec 2;11(12):e0167475. doi: 10.1371/journal.pone.0167475 (PMC5135130; doi:10.1371/journal.pone.0167475)

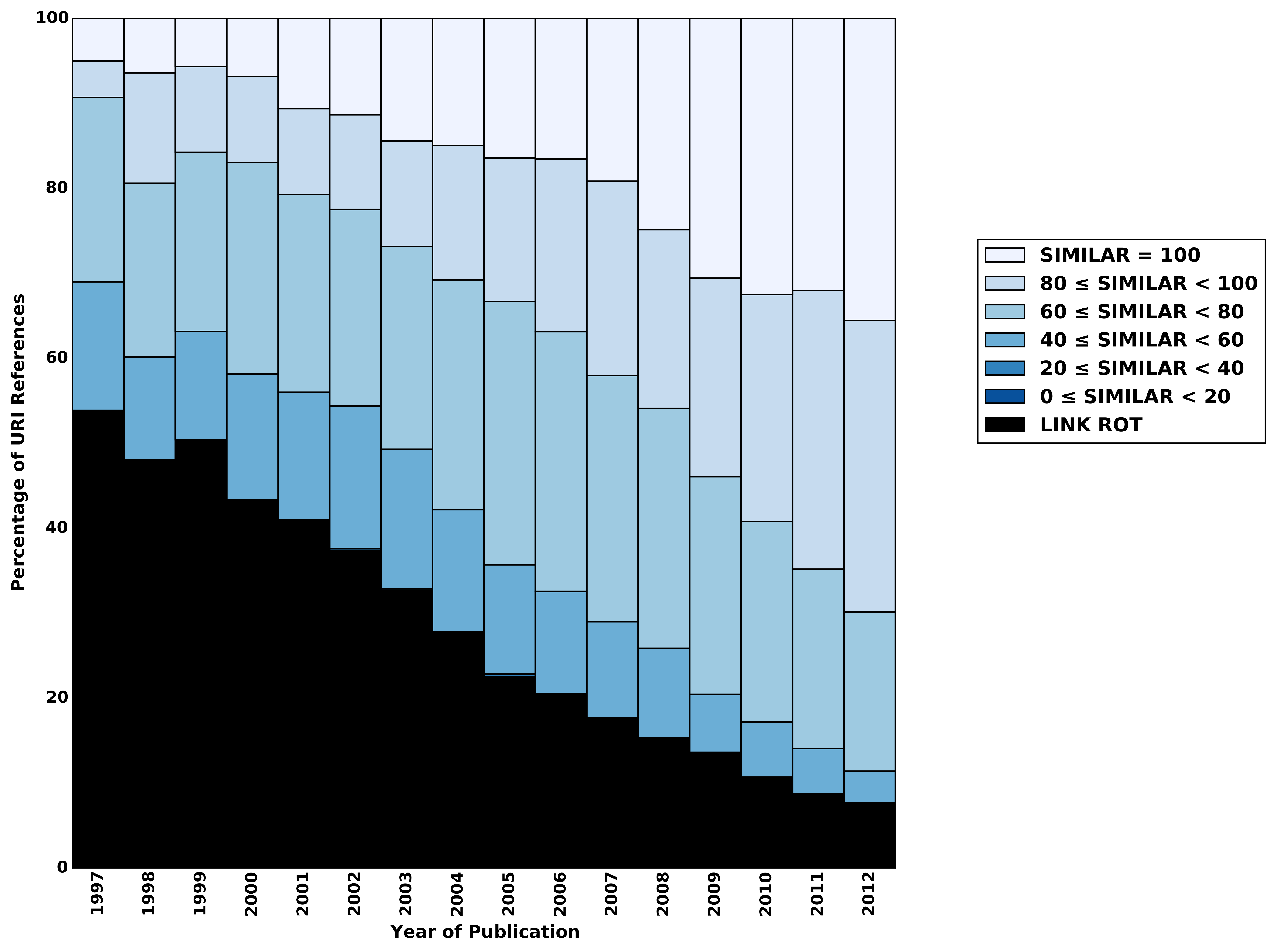

Supplement: S1 Fig — (TIF) [file pone.0167475.s001.tif]

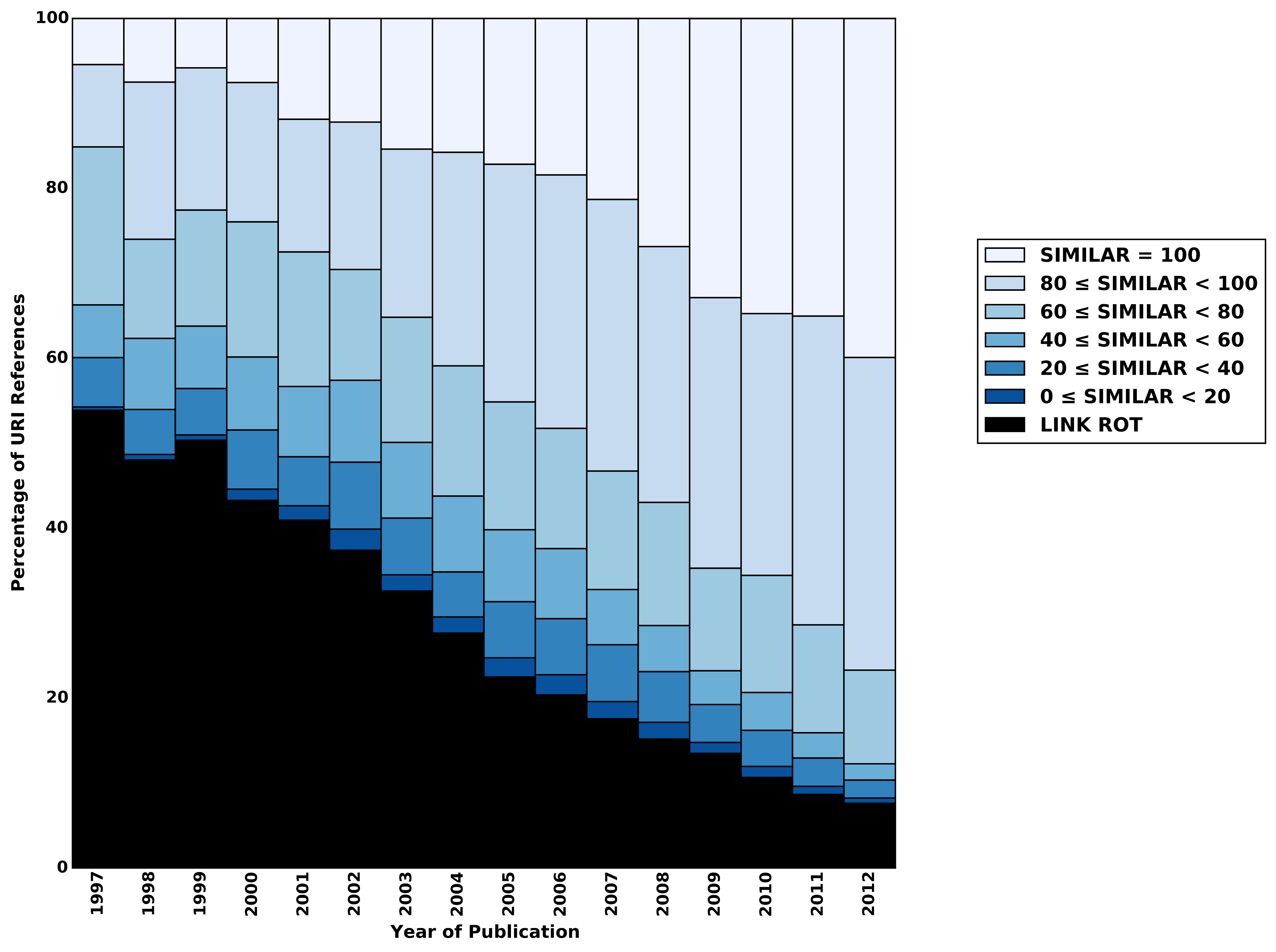

Supplement: S2 Fig — (TIF) [file pone.0167475.s002.tif]

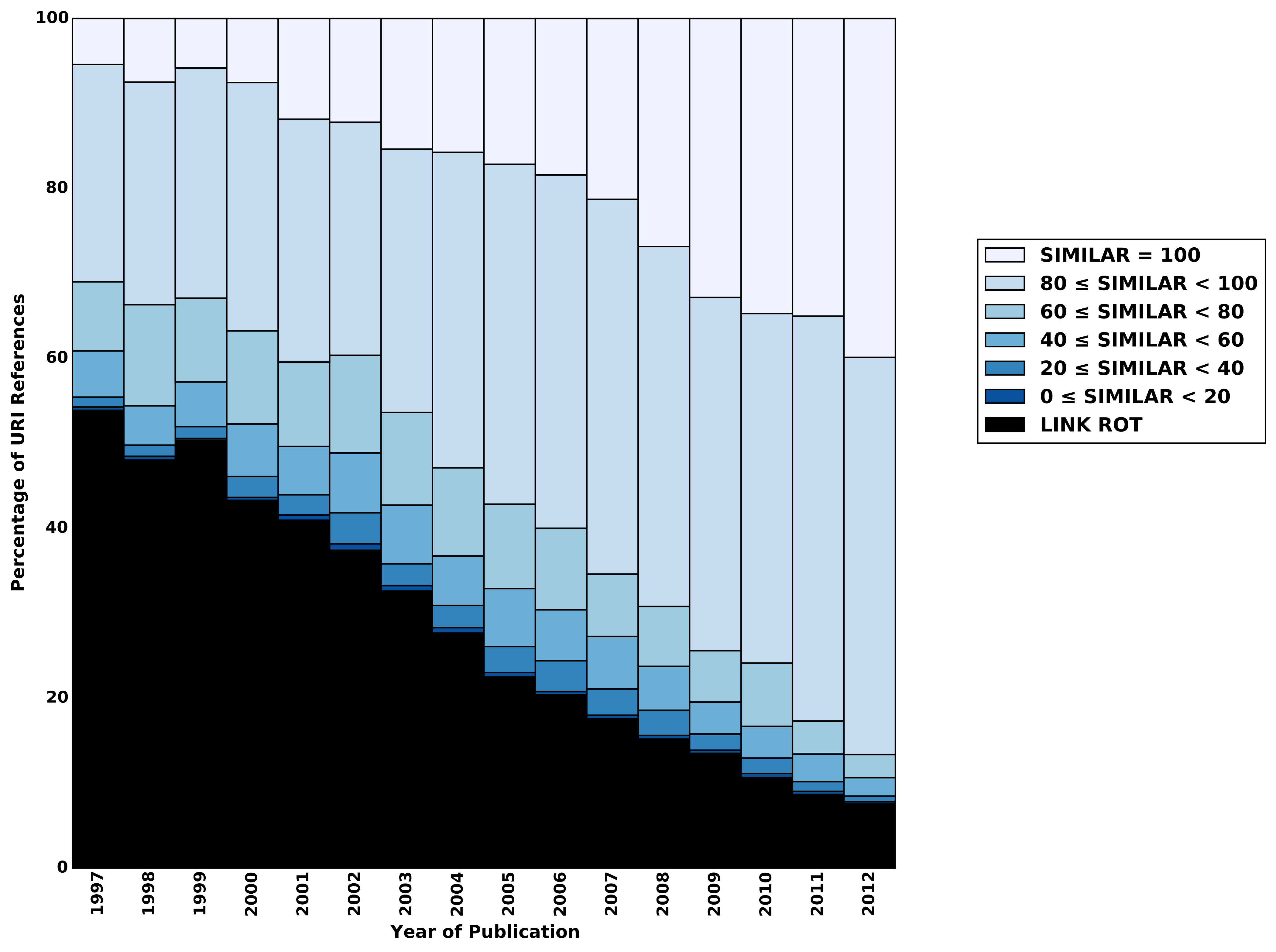

Supplement: S3 Fig — (TIF) [file pone.0167475.s003.tif]

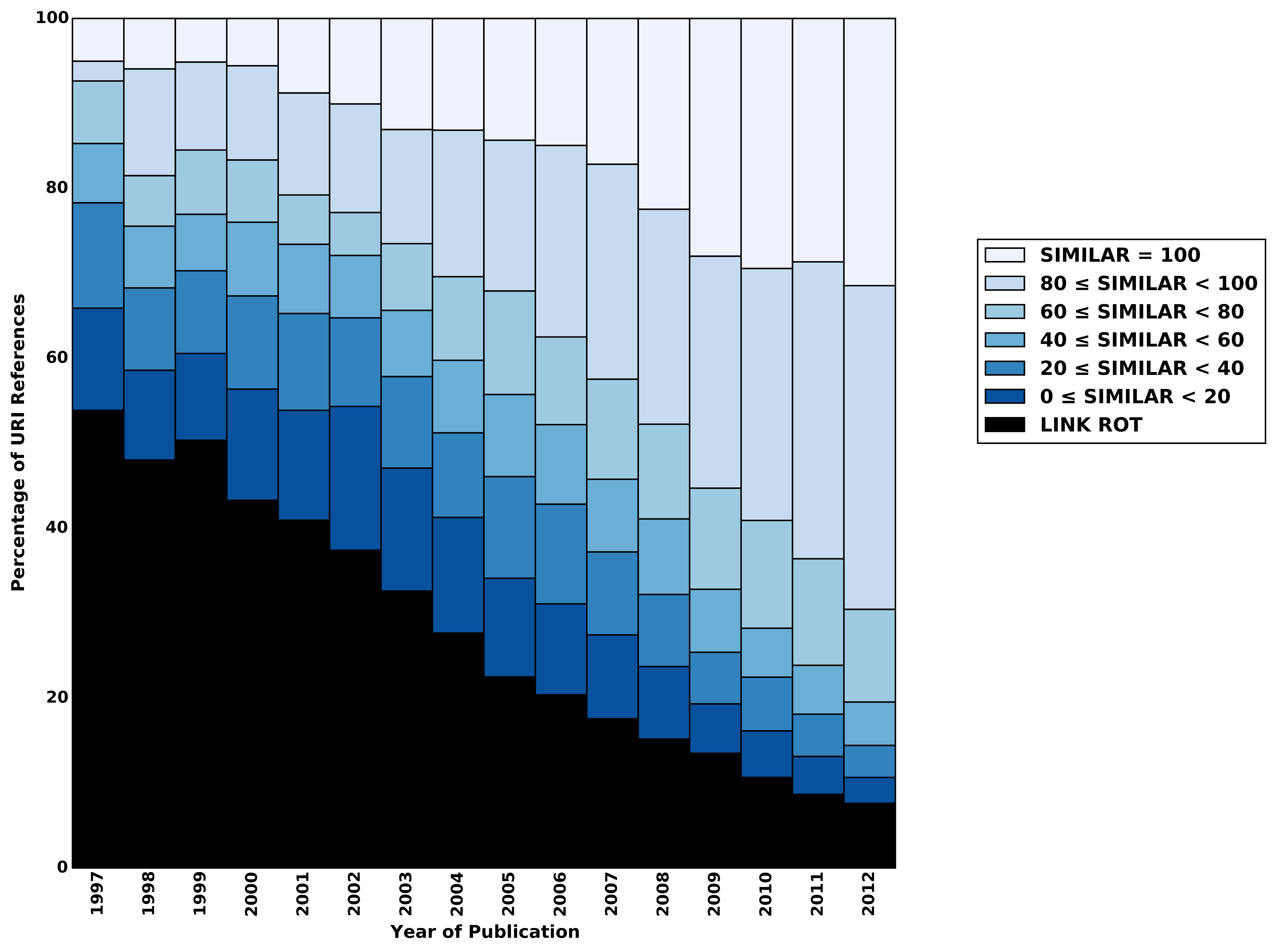

Supplement: S4 Fig — (TIF) [file pone.0167475.s004.tif]

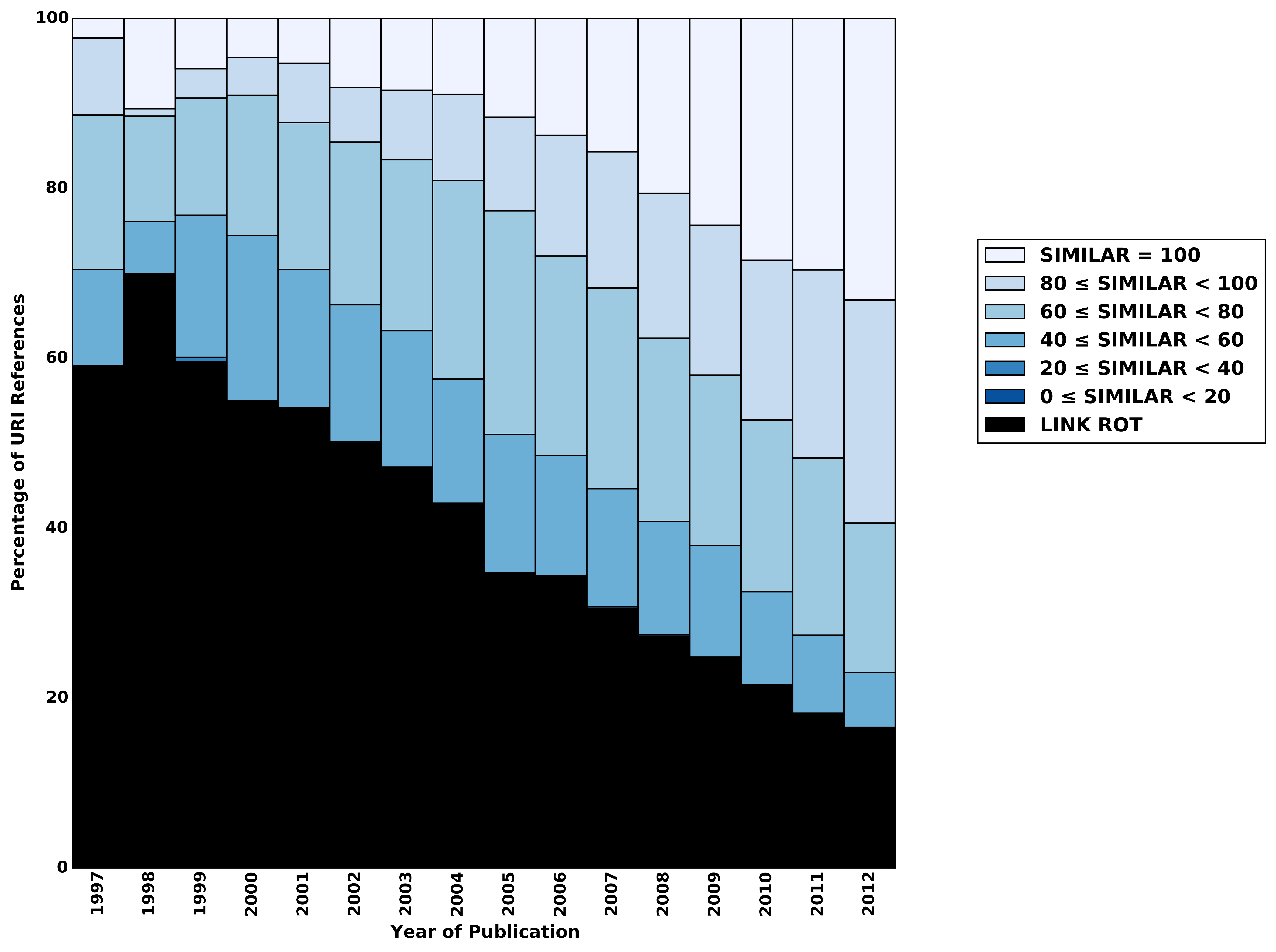

Supplement: S5 Fig — (TIF) [file pone.0167475.s005.tif]

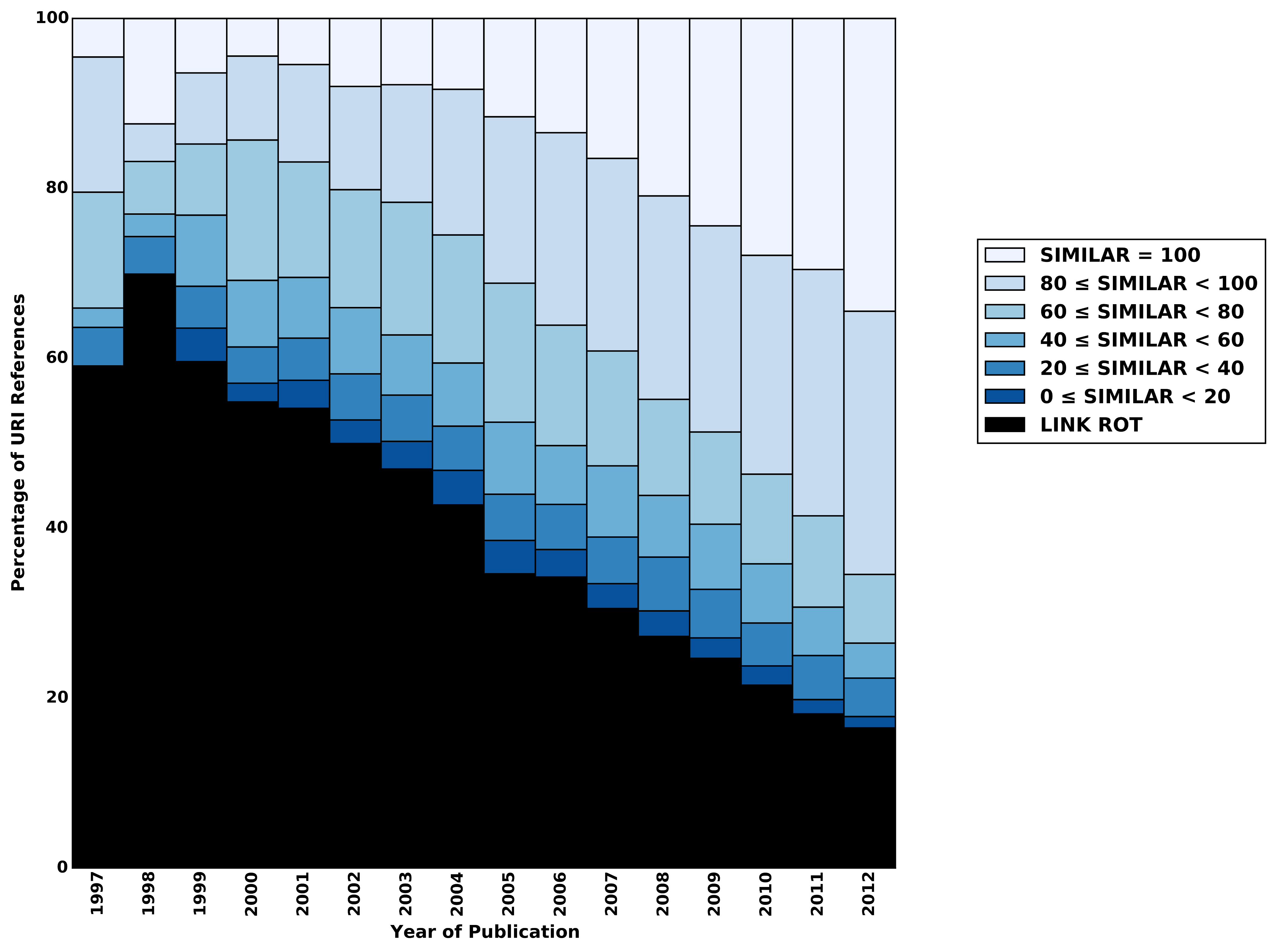

Supplement: S6 Fig — (TIF) [file pone.0167475.s006.tif]

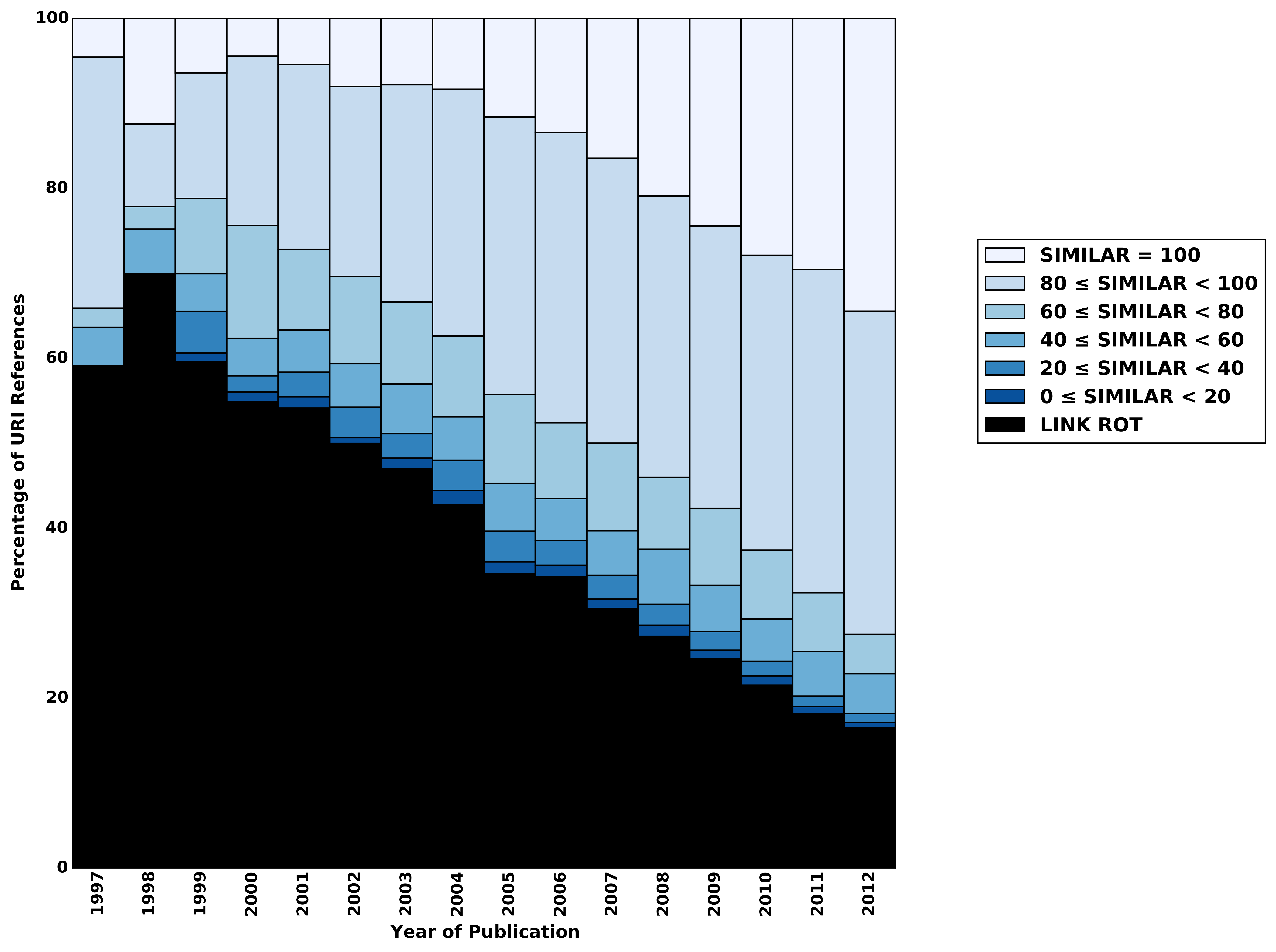

Supplement: S7 Fig — (TIF) [file pone.0167475.s007.tif]

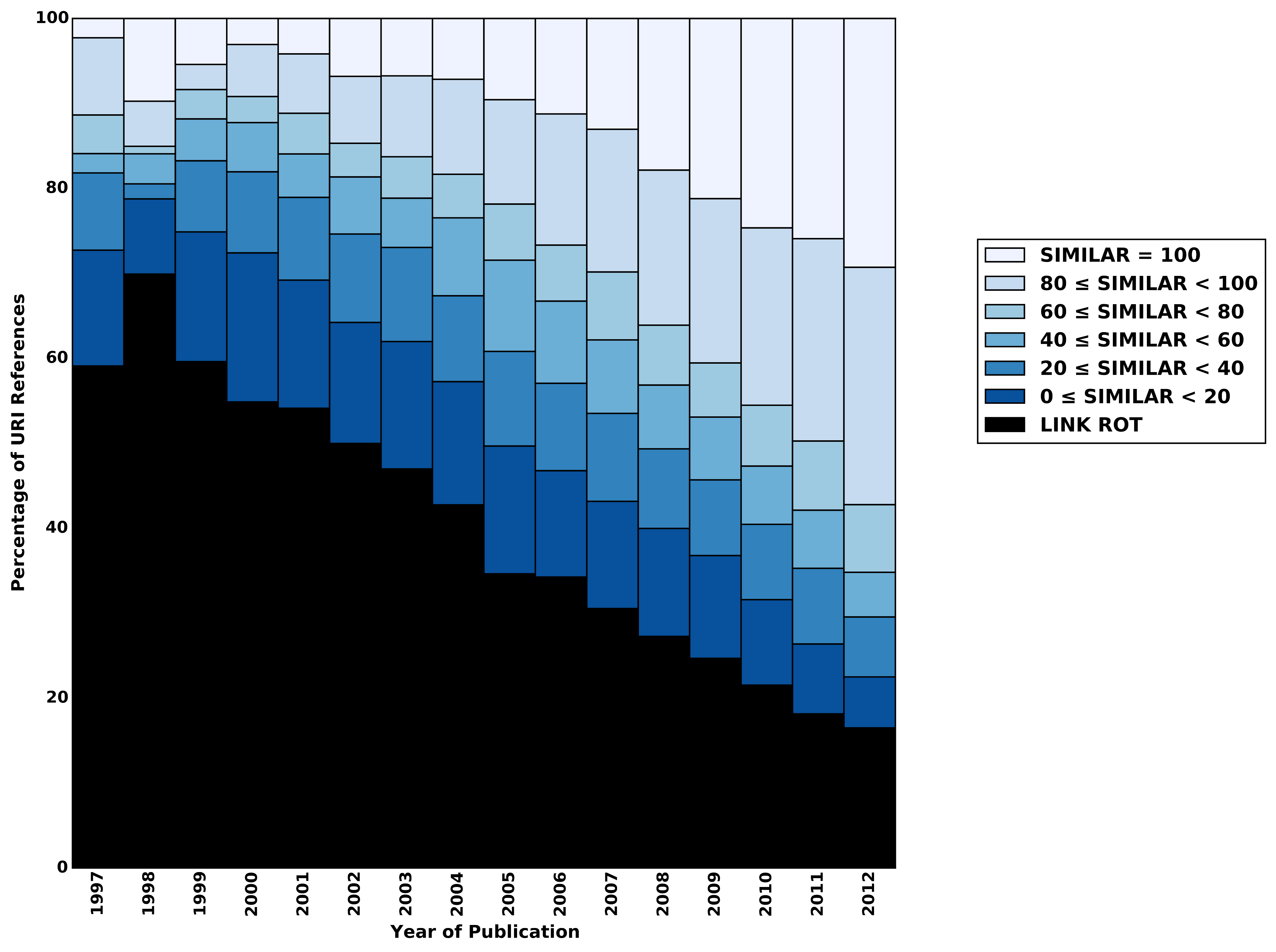

Supplement: S8 Fig — (TIF) [file pone.0167475.s008.tif]

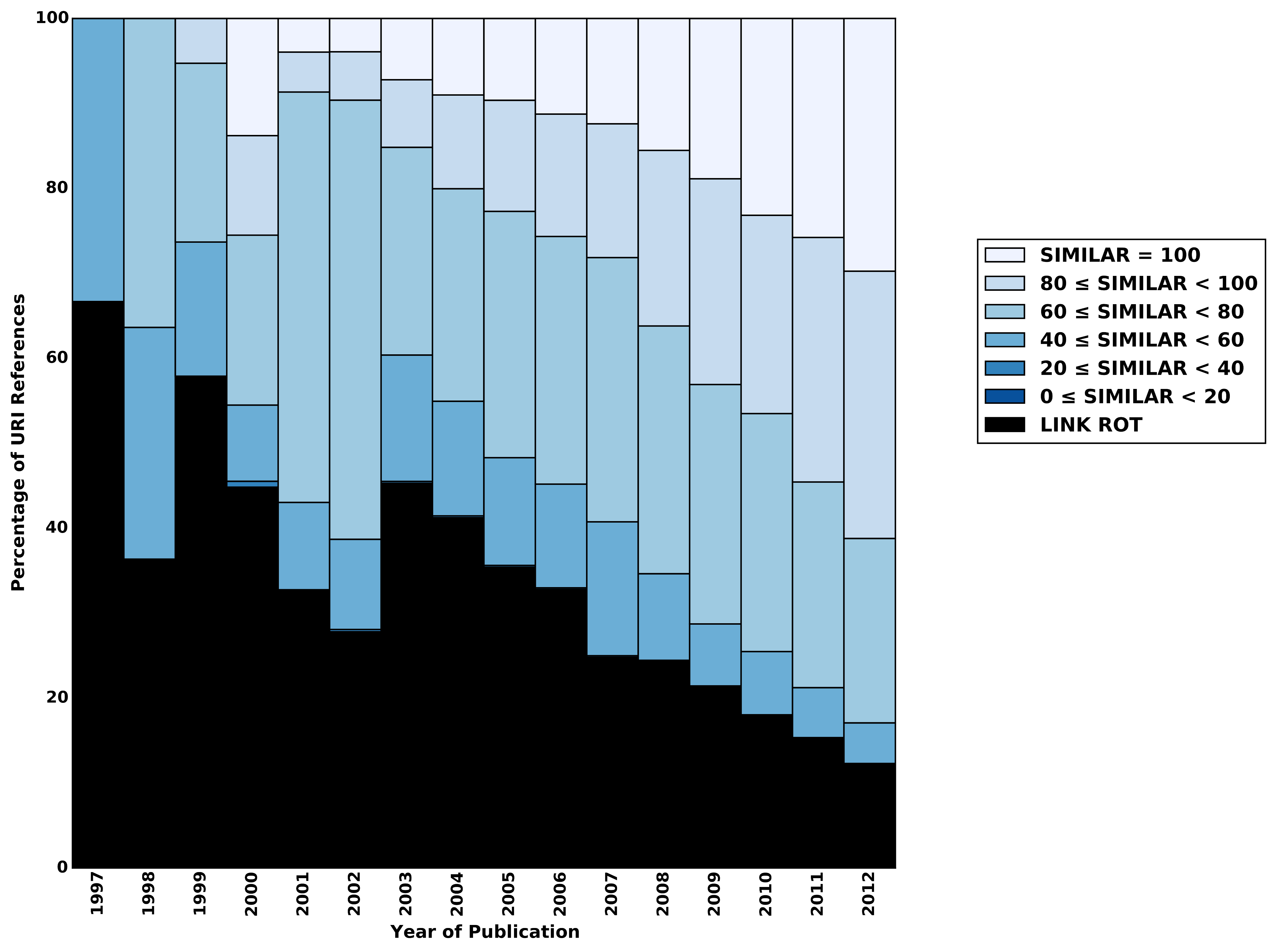

Supplement: S9 Fig — (TIF) [file pone.0167475.s009.tif]

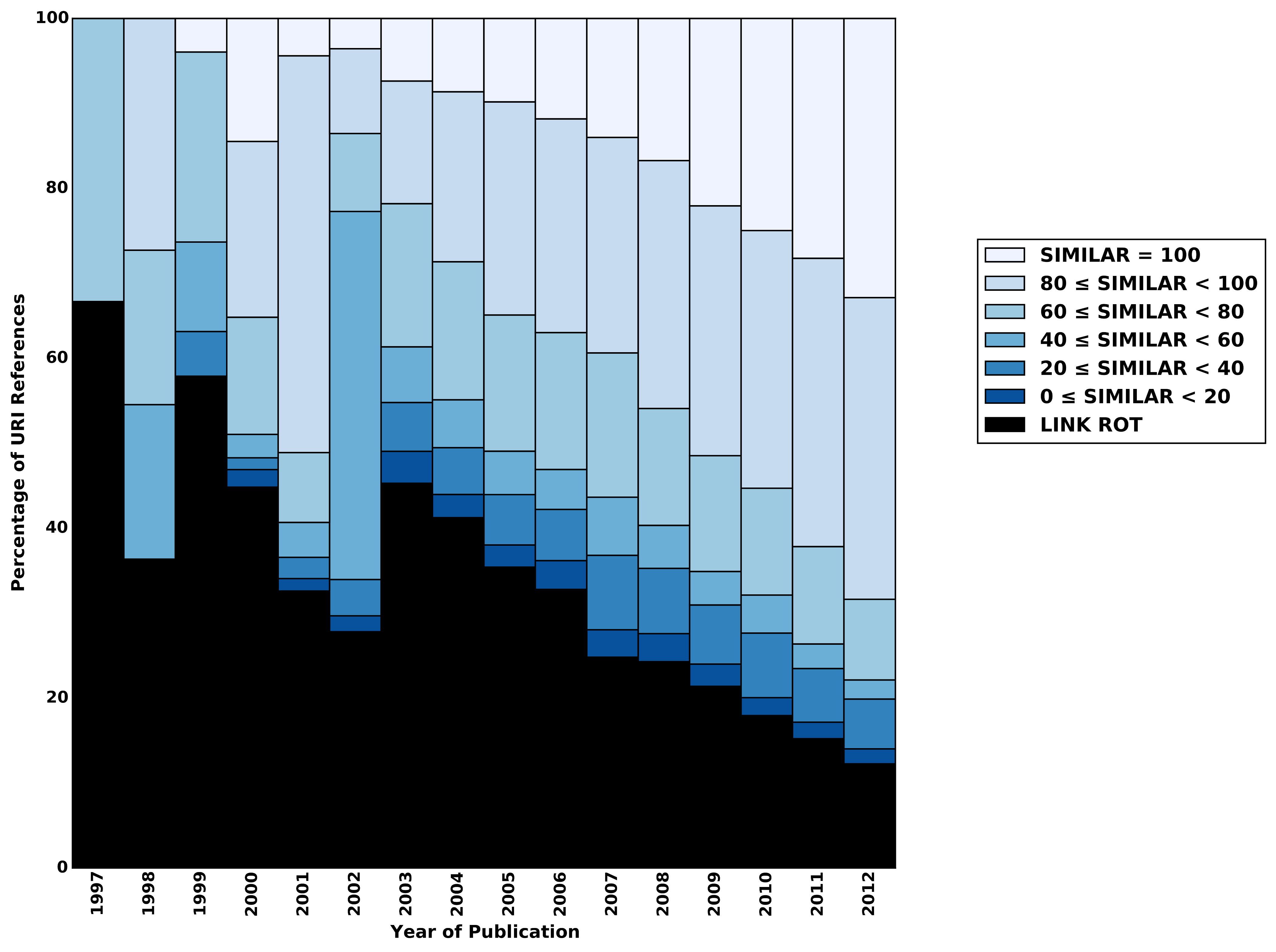

Supplement: S10 Fig — (TIF) [file pone.0167475.s010.tif]

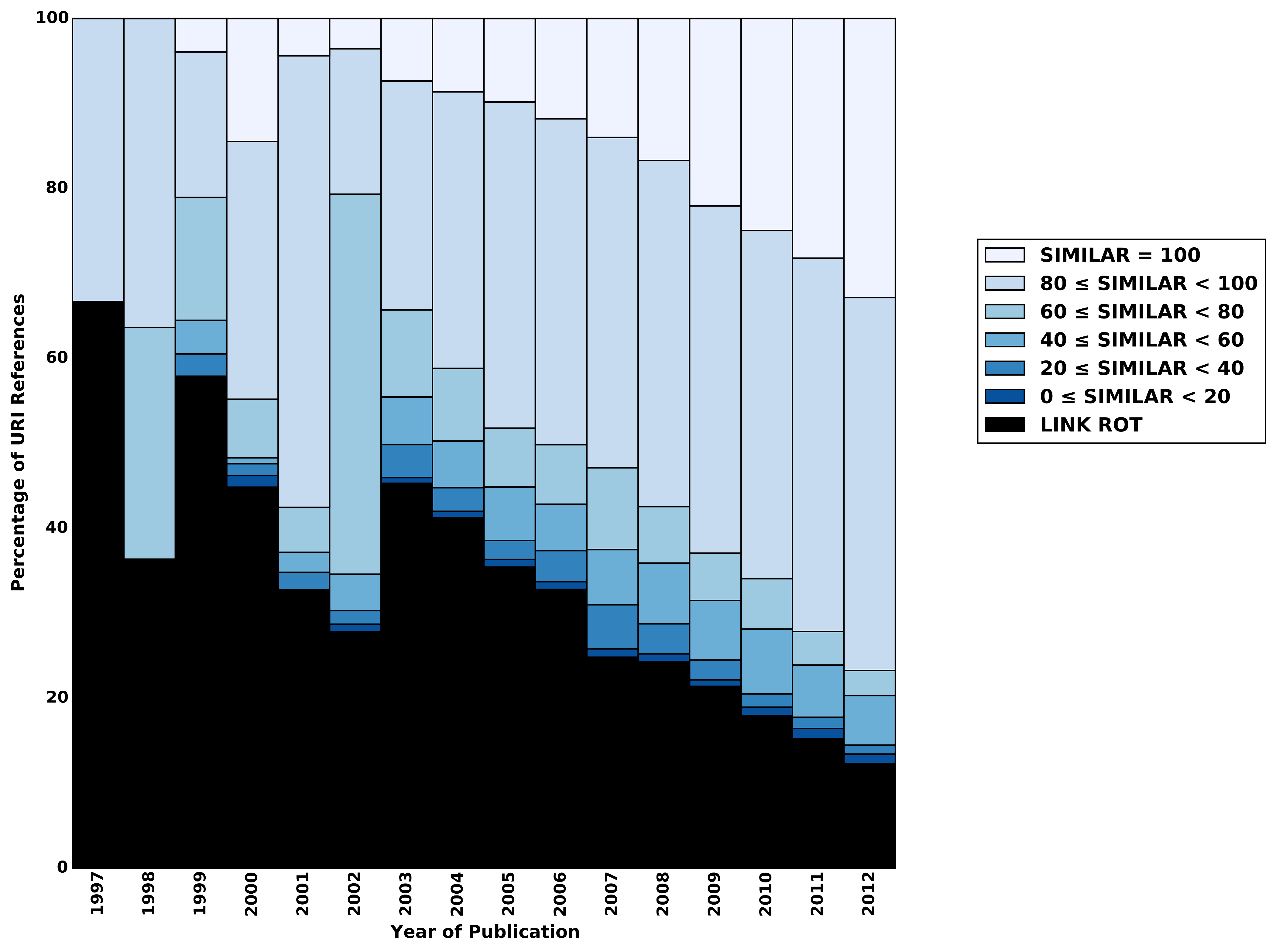

Supplement: S11 Fig — (TIF) [file pone.0167475.s011.tif]

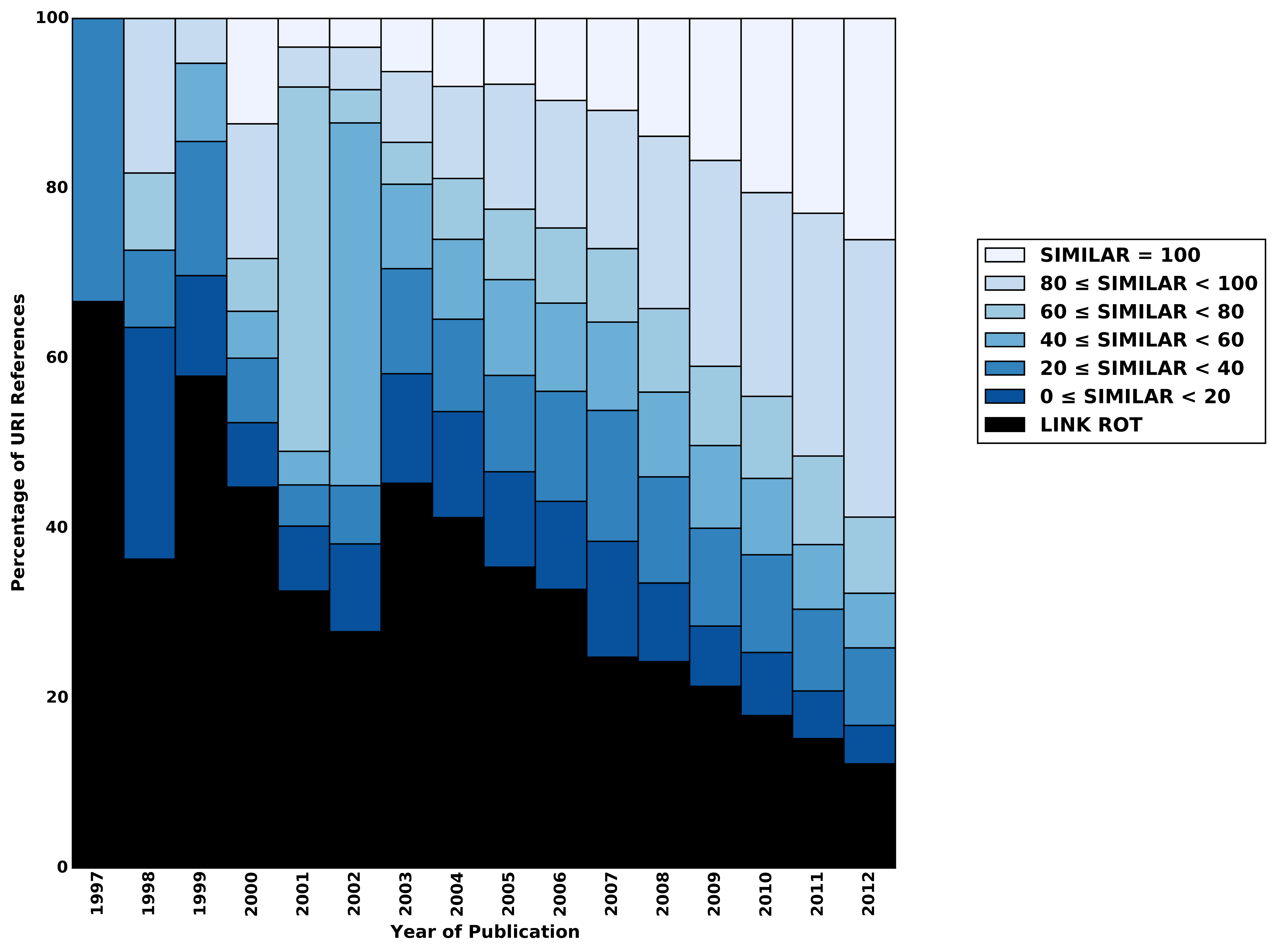

Supplement: S12 Fig — (TIF) [file pone.0167475.s012.tif]
